# WssI from the Gram-negative bacterial cellulose synthase is an *O*-acetyltransferase that acts on cello-oligomers with several acetyl donor substrates

**DOI:** 10.1016/j.jbc.2023.104849

**Published:** 2023-05-22

**Authors:** Alysha J.N. Burnett, Emily Rodriguez, Shirley Constable, Brian Lowrance, Michael Fish, Joel T. Weadge

**Affiliations:** Department of Biology, Wilfrid Laurier University, Waterloo, Ontario, Canada

**Keywords:** microbial biofilms, SGNH hydrolase, glycobiology, acetyltransferase, exopolysaccharide biosynthesis, bacterial cellulose, cellulose synthase

## Abstract

In microbial biofilms, bacterial cells are encased in a self-produced matrix of polymers (*e.g.*, exopolysaccharides) that enable surface adherence and protect against environmental stressors. For example, the wrinkly spreader phenotype of *Pseudomonas fluorescens* colonizes food/water sources and human tissue to form robust biofilms that can spread across surfaces. This biofilm largely consists of bacterial cellulose produced by the cellulose synthase proteins encoded by the *wss* (WS structural) operon, which also occurs in other species, including pathogenic *Achromobacter* species. Although phenotypic mutant analysis of the *wssFGHI* genes has previously shown that they are responsible for acetylation of bacterial cellulose, their specific roles remain unknown and distinct from the recently identified cellulose phosphoethanolamine modification found in other species. Here, we have purified the C-terminal soluble form of WssI from *P. fluorescens* and *Achromobacter insuavis* and demonstrated acetylesterase activity with chromogenic substrates. The kinetic parameters (*k*_cat_/*K*_*M*_ values of 13 and 8.0 M^−1^ s^−1^, respectively) indicate that these enzymes are up to four times more catalytically efficient than the closest characterized homolog, AlgJ from the alginate synthase. Unlike AlgJ and its cognate alginate polymer, WssI also demonstrated acetyltransferase activity onto cellulose oligomers (*e.g.*, cellotetraose to cellohexaose) with multiple acetyl donor substrates (*p*-nitrophenyl acetate, 4-methylumbelliferyl acetate, and acetyl-CoA). Finally, a high-throughput screen identified three low micromolar WssI inhibitors that may be useful for chemically interrogating cellulose acetylation and biofilm formation.

Biofilms are surface-associated microbial communities encased in a self-produced extracellular matrix. The biofilm matrix consists of an array of complex extracellular polymeric substances, which are often comprised of an inventory of biomolecules (*e.g.*, polysaccharides, nucleic acids, proteins, lipids), and their combined structural and functional diversity has recently been termed the matrixome (1). The matrixome provides prominent properties of biofilms, including surface adhesion (*i.e.*, to human tissue, medical devices, fresh fruits and vegetables, other food products, and water sources (2, 3, 4, 5, 6)), as well as a mechanism of defense for physical protection from a variety of environmental stresses (*e.g.*, pH and temperature fluctuations (7), osmolarity (7), shear stress (8), antimicrobial compounds (7, 8), and disinfection agents (9)) and increased protection to immune responses (*e.g.*, antimicrobial peptides, enzyme degradation, and reactive oxygen species) (8, 10). The differences in matrixome composition directly influence the properties that are conferred and are often optimized by community members for specific environmental conditions or niches. For example, the colonization of biotic and abiotic surfaces by some opportunistic human and plant pathogens has been linked to the production of biofilms composed of the β-(1→4)-linked d-glucose polymer, cellulose. A growing list of bacteria, including *Acetobacter* (11, 12), *Clostridium* (previously *Sarcina*) (6, 11), *Bordetella* (13), *Komagataeibacter* (13), *Rhizobium* (11), *Agrobacterium* (11), *Cronobacter* (5), *Salmonella* (14), *Escherichia coli* (15, 16, 17), and *Pseudomonas* (18), are now known to produce cellulose and are of clinical and economical importance. Of the organisms on this list, *E. coli* and *Salmonella enterica* are the most extensively studied with respect to bacterial cellulose biosynthesis and have served as model organisms for understanding the synthesis and export of the polymer (3, 16, 17, 19). In these two model organisms, microbial cellulose is decorated by the transferase BcsG with phosphoethanolamine, which is predicted to aid in resistance to cellulases because of altered phenotypic effects when the decorated cellulose fibrils are part of the biofilm along with amyloid curli fibers (15, 20, 21, 22).

The modification of secreted exopolysaccharides within the matrixome has also been observed in other biofilm model systems as a mechanism for bacterial adaptation to diverse environments (23). The acetylated alginate pathway, conferred by translation of the *alg* operon, is a synthase-dependent system that is best characterized in the human pathogen *Pseudomonas aeruginosa* and is associated with severe bacterial biofilm-related infections in the airway of cystic fibrosis and SARS-CoV-2 infected patients (24, 25, 26, 27). The acetylated alginate biofilm has challenged traditional treatment methods because of its recalcitrant properties and has emerged as a significant clinical area of study. Other Pseudomonads, such as *Pseudomonas fluorescens*, do not produce alginate but do exhibit similar acetylation patterns with respect to the production of microbial cellulose instead (18, 23). Spiers *et al.* (18) were the first to demonstrate that these strains can acetylate either the 2, 3, or 6 carbon positions of individual glucose residues that are β-1,4-linked into a cellulose polymer with an overall acetylation level of 14%. Gene clusters with significant sequence similarity to the acetylation machinery loci of alginate have also been identified within the bacterial cellulose operon. This gene synteny suggests that cellulose acetylation likely coevolved alongside or from corresponding alginate operons in *Pseudomonas* species (23). *P. fluorescens* is often considered to be less pathogenic than *P. aeruginosa* but maintains clinical relevance as this pathogen persists in infected organs and tissues of humans (*e.g.*, the uterus, urinary tract, skin, sinus, lungs, eye, cerebrospinal fluid, bone, and blood) (28). In addition, *P. fluorescens* is a known food-related pathogen that causes spoilage of pasteurized liquid milk products, fruits, vegetables, and meats, which further outlines this species of *Pseudomonas* as an emergent threat in need of further investigation to mitigate its spread and colonization of products intended for human consumption (29).

Often misidentified as *P. aeruginosa*, *Achromobacter* species have also been noted to produce exopolysaccharides, including cellulose (30), within their matrixome, and some strains possess a complementary set of genes with high homology to those that produce acetylated cellulose in *P. fluorescens*. Possibly accounting for some of the misidentification with *Pseudomonas*, *Achromobacter* infections are most commonly noted in the respiratory tract, with strong biofilm production occurring in the sputum of cystic fibrosis patients in nosocomial environments (31, 32). However, *Achromobacter* infections have also been identified in other areas of the human body, such as the urinary tract, ulcers, wounds, inner wall of the abdomen, spinal cords, bones, and prosthetic implants (31, 32, 33, 34, 35, 36). Of further relevance, *Achromobacter* is the predominant colonizer of contact lenses, solutions, and cases leading to bacterial-related keratitis of the eye and has been isolated from the soil as well as domestic (hospital) and environmental aquatic sources (33, 37, 38, 39, 40). Although mostly noted for their unfavorable clinical association, colonization by *P. fluorescens* and *Achromobacter* species can lead to advantages for some hosts. Following colonization that is often biofilm based, both organisms have been identified to suppress plant wilting (*i.e.*, in tomato, wheat, and tobacco) by producing antibiotic compounds to defend against fungal pathogens (41, 42). Taken together, the diverse implications of *P. fluorescens* and *Achromobacter* spp. on a variety of hosts has established a valuable opportunity to use these organisms as models to broaden our understanding of modified cellulose synthase–dependent systems within these new settings, while also expanding our knowledge beyond cellulose phosphoethanolamine modifications to include those that employ acetylation as well.

To date, our knowledge of cellulose acetylation in microbes is largely still based on work with *P. fluorescens.* The genetic map of the *P. fluorescens* SBW25 prototype strain was originally identified by Rainey and Bailey in 1996 (43). Subsequent studies using *in vitro* expression technology concluded that *P. fluorescens* underwent divergent evolution through adaptation to a new niche; thereby causing development of a mutant with a new phenotype that has a significant fitness advantage over the ancestral strain (44, 45). This new mutant of *P. fluorescens* produced a strong biofilm pellicle at the air–liquid interface of growth media (noted as the Wrinkly Spreader phenotype) that increased the fitness of the microbe, enhanced the spreading of the biofilm, and improved adherence to surfaces (44). The genes responsible for this phenotype were identified through transposon mutagenesis by Spiers *et al.* (18, 46, 47, 48, 49) to belong to an operon that they termed WS structural (*wss*), which when expressed, increased the virulence of this opportunistic species by overproducing acetylated cellulose, likely acting as a cohesive factor in concert with fimbrial-like components to control surface colonization.

The *wss* operon encodes a synthase-dependent system of proteins responsible for both the synthesis and acetylation of cellulose containing biofilms. The gene products of *wssA-J* share homology with genes from the cellulose (*bcsABZC*) and alginate (*algX/FIJ*) pathways (noted in Fig. 1) (18, 46, 48, 50). Regulation of cellulose biosynthesis is thought to occur through the encoded WssB and WssC proteins that are initiated by binding of the secondary messenger bis-(3′,5′)-cyclic dimeric guanosine monophosphate to coordinate the polymerization and concomitant transport of the polymer across the inner membrane, which has been proven for their BcsA/B homologs (16, 17, 46, 51). Predicted to function like BcsC, WssE is hypothesized to guide the polymer through the periplasm and facilitate export across the outer membrane (46, 50, 52). WssD is a predicted glycoside hydrolase, like BcsZ from *E. coli* and CcsZ from *Clostridium difficile*, and is proposed to cleave the growing polymer to allow release from the cell into the biofilm matrix (46, 50, 53, 54). While the downstream *wssFGHI* genes (or homologs thereof) are beginning to be noted with cellulose synthase systems, the biochemical activity of the individual gene products remain uncharacterized (12, 55). However, these gene products share homology to *algX/FIJ*, which contribute to acetylation of alginate in *P. aeruginosa*, so *wssFGHI* are likewise predicted to encode for proteins that decorate the cellulose polymer with acetyl groups in an analogous fashion. By this model, acetyl groups are transferred into the periplasmic space from a donor protein (WssH) and attached onto microbial cellulose by the concerted action of WssFGI prior to export of the modified polymer from the cell (46). Finally, WssA and WssJ (not shown in Fig. 1) are homologous to BcsQ and MinD from *E. coli* and are proposed to be responsible for correct spatial localization of the complex in the cell (46, 56).

**Figure 1:**
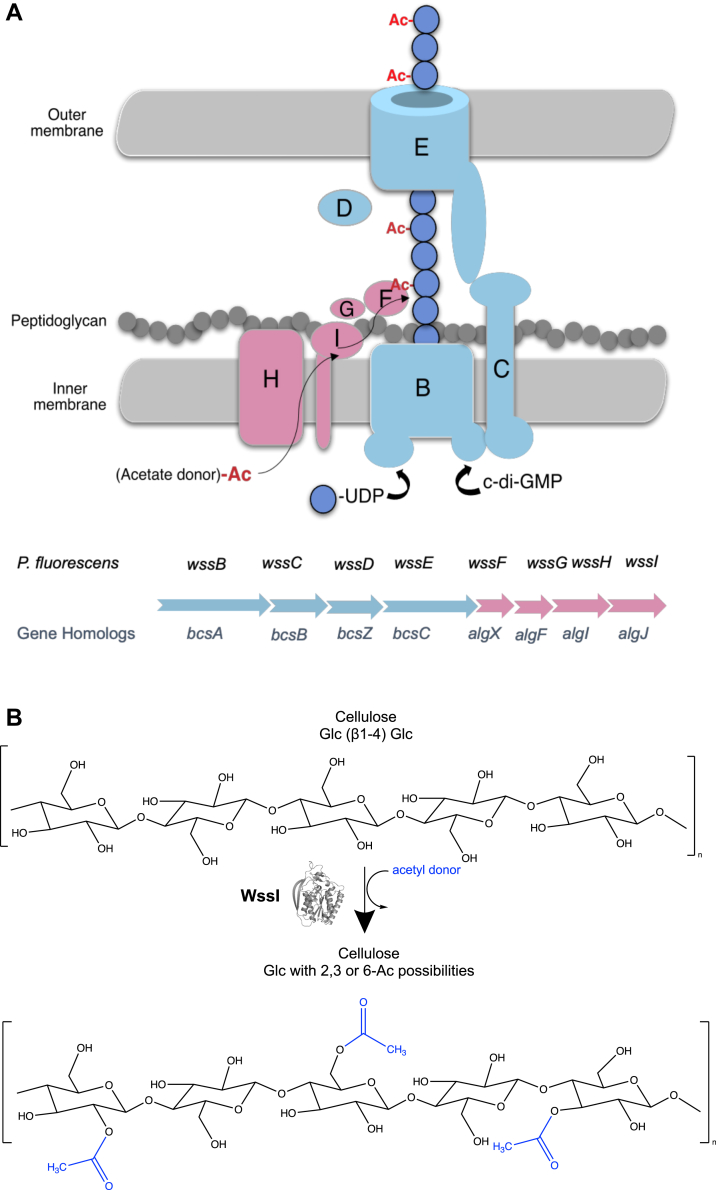
**Schematic representation of the acetylated-cellulose exopolysaccharide synthase–dependent system and operon structure.***A*, organization of the *wss* operon in *Pseudomonas fluorescens* is indicated below the diagram of the predicted synthesis apparatus for reference. *Light blue* genes and proteins are homologous to the Bcs proteins responsible for cellulose synthesis by UDP-glucose polymerization and export of newly synthesized chains (represented as *blue circles*). *Pink* genes and proteins are homologous to the alginate (*alg*) modification enzymes that transfer acetyl groups from an unknown donor into the periplasmic space and onto the cellulose polymer by a concerted action of WssFGHI prior to export. *B*, proposed enzymatic acetyltransfer onto a cellulose polymer performed by WssI at one of either the 2, 3, or 6 carbon positions of glucose to an overall level of 14% across the polymer (18). Figure adapted from prior acetylated-exopolysaccharide models (50, 54, 81).

While the function of the Wss proteins involved in the acetylation and secretion of cellulose can be predicted based on homology, Spiers *et al.* (18, 46, 48) were also able to demonstrate the effects of *wssFGHI* mutants *in vivo* on *P. fluorescens* biofilms. Individual mutants of *wssF* and *wssI* demonstrated a unique smooth colony phenotype compared with that of the Wrinkly Spreader cellulose-producing strain that was given its name based on its colony appearance on plates. The prominent biofilm layer at the air–liquid interface formed by the mutants was easily disrupted despite the fact that cellulosic material was still overproduced by this strain (18). In separate studies, the *bcsII* (type 2) operon in *Komoagataeibacter xylinus* was similarly shown to be involved in the colonization of the air–liquid interface, and sequence comparisons suggest that this operon also possesses genes (*i.e.*, labeled *bcsXYZ*) that influence the levels of cellulose acylation (12, 55). These combined results support that WssFGHI work in a concerted fashion to acetylate the cellulose polymers, which promotes biofilm/colony spreading, adherence to surfaces, and maintenance of a robust biofilm that, at least in *P. fluorescens*, contributes to virulence (18, 46, 48). Further *in vitro* work of the gene products is now required to understand their direct function within the acetylated cellulose pathway of these organisms and the role of the resultant polymer in the matrixome.

Herein, we present the first biochemical characterization of the C-terminal catalytic domain of WssI from *P. fluorescens* (*Pf*WssI^ΔN^) and *A. insuavis* (*Ai*WssI^ΔN^). While these enzymes were previously predicted to be part of the cellulose O-acetylation machinery for these bacteria, we demonstrate for the first time that the C-terminal soluble forms of these WssI proteins (WssI^ΔN^) are indeed acetyltransferases capable of transferring acetyl groups from a variety of donor substrates onto cello-oligosaccharides (Fig. 1). We also outline the structural and catalytic features of this enzyme that place it in the SGNH hydrolase family. Armed with knowledge of the reaction catalyzed by WssI^ΔN^, extensive high-throughput screening (HTS) was employed to identify the first inhibitors of this cellulose acetyltransferase that now permit their use as enzymatic tools to better understand the cellulose synthase pathway. Together, this work provides the foundation for how WssI contributes to bacterial cellulose acetylation within *P. fluorescens* and *A. insuavis*, two organisms of clinical, environmental, and economical importance.

## Results and discussion

### WssI is a member of the SGNH family of proteins

**Figure 2:**
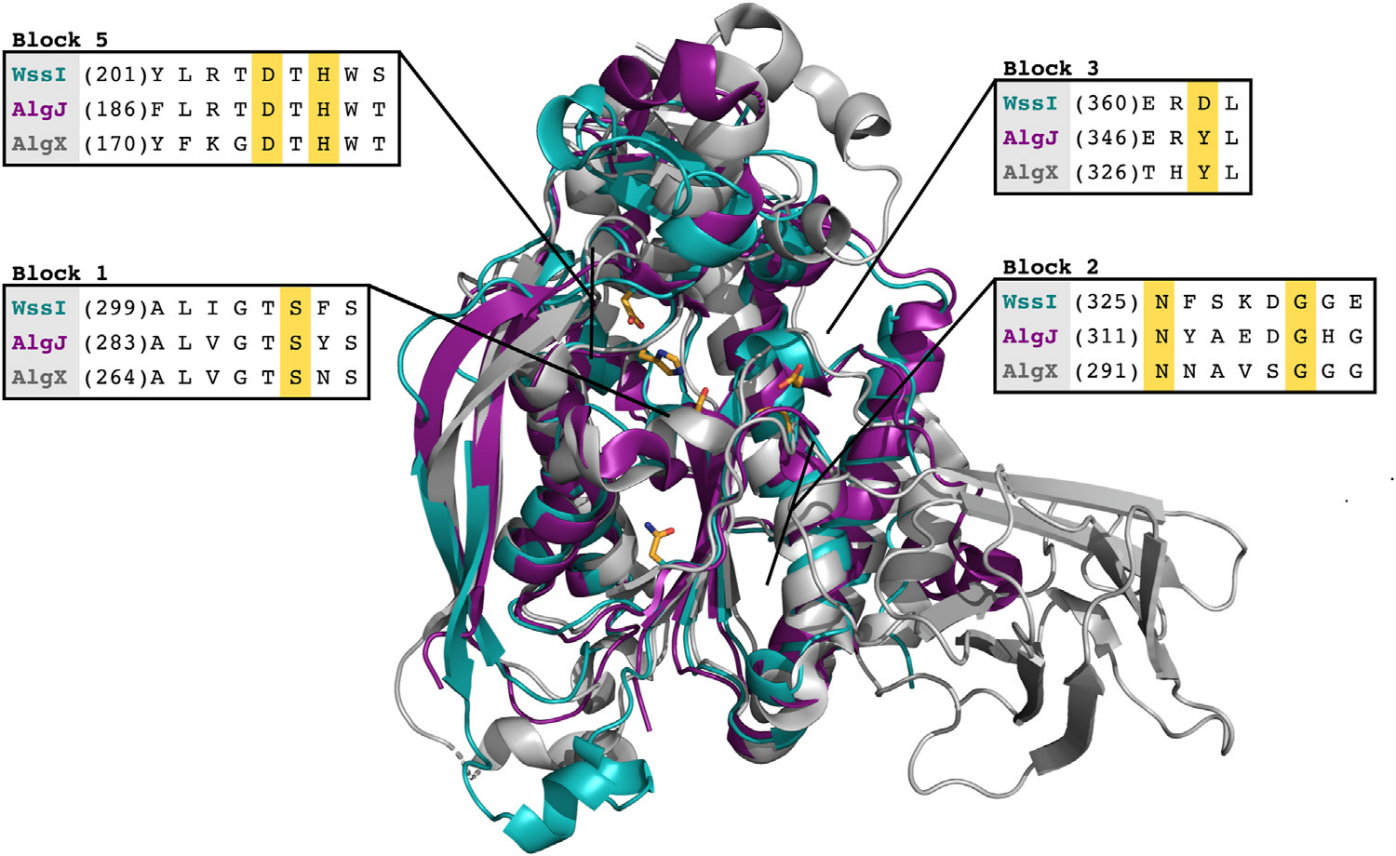
**Sequence alignment analysis of WssI**^**ΔN**^**with the AlgJ and AlgX homologs.** Structural overlay of the AlphaFold2 predicted structure of *Pf*WssI^ΔN^ (*cyan*), *Pseudomonas putida* AlgJ (Protein Data Bank ID: 4O8V) (*purple*), and *Pseudomonas aeruginosa* AlgX (Protein Data Bank ID: 4KNC) (*gray*). Phyre2 sequence alignments of *Pf*WssI^ΔN^ have 73% confidence and 29% amino acid identity with AlgJ and 72% coverage and 25% amino acid identity with AlgX. The conserved SGNH family residues, including the active site serine, histidine, and aspartate residues, are shown in *yellow*. The structural image was rendered in PyMol, and the alignment was performed with Clustal Omega. The root mean standard deviation of *Pf*WssI^ΔN^ and AlgJ is 1.25 Å (across 1236 Cα atoms) and 2.71 Å (across 1166 Cα atoms) between *Pf*WssI^ΔN^ and AlgX (which excluded the C-terminal carbohydrate-binding domain of AlgX). *Pf*WssI, WssI from *Pseudomonas fluorescens*.

The active sites of SGNH hydrolases, including esterase, lipase, and transferase members, participate in a proton relay that activates a catalytic serine for nucleophilic attack on incoming ester-linked substrates (57, 58, 59). Amongst identified SGNH hydrolase homologs of WssI that have been characterized, the AlgJ, AlgX, and PatB1 proteins have the highest relatedness to *Pf*WssI, with 29, 25, and 14% amino acid sequence identity, respectively, and similarity ranging between 45 and 47% (outlined in Table 1) across the SGNH hydrolase regions. All homologs are acetylesterases or acetyltransferases that are responsible for modifying carbohydrate polymers outside the bacterial cell. AlgJ and AlgX from *P. aeruginosa* are involved in the acetylation of alginate, whereas PatB1 is a secondary cell wall polysaccharide (SCWP) *O-*acetyltransferase from *Bacillus cereus* (60, 61, 62). Notably from the primary structure, the resemblance of WssI to AlgJ goes beyond the core SGNH hydrolase features. Both proteins are predicted type II membrane proteins containing an N-terminal signal sequence that is retained as a transmembrane protein anchor. The order of the amino acids found in conserved primary structure blocks 1, 2, 3, and 5 (corresponding to the location of the S-G-N-H residues originally identified by Akoh *et al.* and for which this family of enzymes are named) is circularly permuted and slightly altered for both AlgJ and AlgX (H-S-G-Y) as well as the WssI proteins (H-S-G-D) (Figs. 2 and S1) (61, 63). This orientation differs from that of the recently identified SGNH domains of *BcsX* and the C-terminal domain of *BcsZ* that are proposed to be involved in acylated cellulose in *K. xylinus* E25, which may be why the sequence identity is so low between these proteins (*i.e.*, less than 20%) (12).

**Table 1:** Alignment parameters from Phyre2 analysis of homologous alginate proteins with *Pf*WssI

Protein homolog (Phyre2 designation)	Coverage (%)^a^	Amino acid identity^b^ (%)	Amino acid similarity^c^ (%)
AlgJ (esterase/transferase)	73	29	47
AlgX (sugar binding/transferase)	71	25	46
PatB1 (*O-*acetyltransferase)	72	14	45

a Percentage of sequence that was covered in the alignment between the two proteins.

b Percentage identity between the input sequence and the template (83).

c Percentage of similar amino acids (*i.e.*, charge, aromatic, etc.) within the aligned sequence (https://www.ncbi.nlm.nih.gov/).

**Figure 3:**
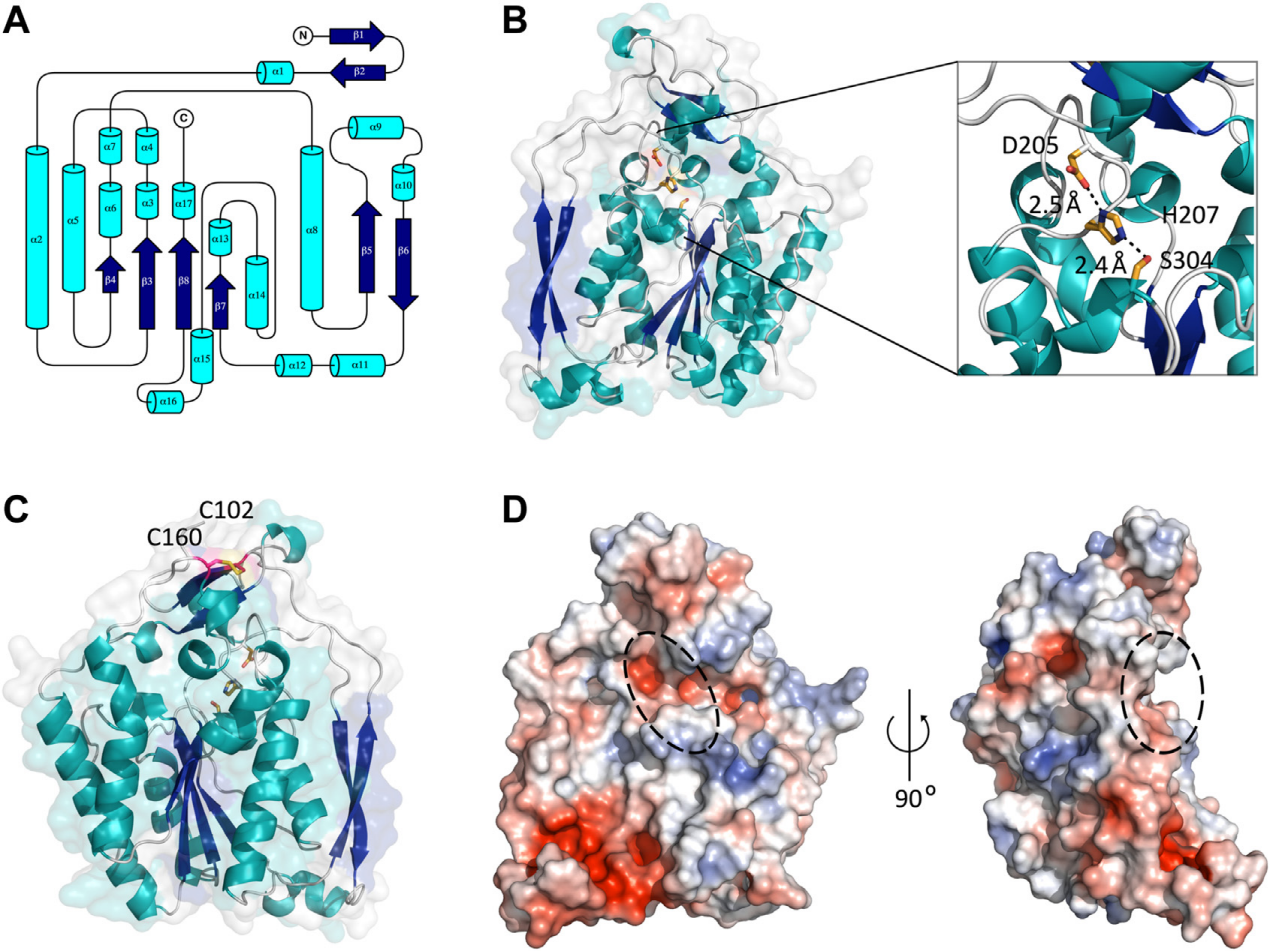
**Predicted structural model of *Pf*WssI**^**ΔN**^**.***A*, two-dimensional topology model of the overall fold of *Pf*WssI^ΔN^. *B*, *Pf*WssI^ΔN^ modeled on the structure of AlgJ from *Pseudomonas putida* (Protein Data Bank ID: 4O8V; 29% amino acid identity) displaying the orientation of predicted catalytic residues Asp205, His207, and Ser304 (depicted in *yellow*). Distances between atoms of active site residues involved in catalysis are shown by *dashed lines* (computed in PyMol). *C*, location of a potential disulfide bond formed by highlighted cysteine residues, Cys160 and Cys102 (shown in *pink*). *D*, orthogonal views of the electrostatic surface potential of *Pf*WssI^ΔN^ that display electronegative regions adjacent to either side of the catalytic triad (within the *dashed circle*). Electrostatics are colored from *red* (−5 kT/e) to *blue* (+5 kT/e) for electronegative to electropositive, respectively. Image files were rendered from AlphaFold2-generated structures in PyMol or TopDraw. *Pf*WssI, WssI from *Pseudomonas fluorescens*.

Homology models of *Pf*WssI^ΔN^ and *Ai*WssI^ΔN^ were generated and have provided further valuable input into key structural features of these proteins. Given that both proteins are highly homologous (62% amino acid similarity) and adopted the same fold (as assessed with bioinformatics predictions and further corroborated with CD analysis of the purified WssI^ΔN^ proteins; Fig. S2), *Pf*WssI^ΔN^ was chosen to highlight the specific features of these proteins for simplicity. *Pf*WssI^ΔN^ is predicted to have four parallel central β-strands flanked by six α-helices in an α/β/α topology that is consistent with other related SGNH esterase and transferase proteins (Figs. 2 and 3). In addition, *Pf*WssI^ΔN^ has a small α/β region modeled above the active site, which is known as the cap and is also found in AlgJ, AlgX, PatB1, a related xylan *O*-acetyltransferase (XOAT1) and an uncharacterized protein from *Eubacterium siraeum* (Protein Data Bank IDs: 408V, 4KNC, 5V8D, 6CCI, and 4NZK, respectively), that plausibly influences access to the substrate-binding pocket. *Pf*WssI^ΔN^ is also predicted to have two long antiparallel β-strands that span the length of one side of the protein, which is a feature noted in the alginate synthase proteins (AlgX and AlgJ) as well, but is not common across all SGNH hydrolases (*e.g.*, not found in XOAT1) (60, 61, 64). In *Pf*WssI^ΔN^, there are two antiparallel β-strands in the cap region that are bracketed by two cysteine residues (Cys160 and Cys102), which may form a disulfide bond (Fig. 3*C*). Disulfide bonds have been noted in the structures of AlgX, PatB1, and XOAT1, but the role of these residues and the cap region are still unknown across these homologs (60, 62, 64). The structural models of WssI^ΔN^ also support the consensus orientation of the proposed active site residues (Ser304, His207, and Asp205 according to numbering for WssI from *P. fluorescens*) to participate in a proton relay that activates the nucleophilic serine, despite the circularly permuted reorganization relative to other SGNH hydrolases (Figs. 2 and 3*B*). This catalytic triad is positioned in the middle of a predicted electronegative cleft across the face of the enzyme, which likely forms the basis of the polysaccharide-binding site (Fig. 3*D*) that is similar in charge and depth to AlgJ (*i.e.*, shallow electronegative groove) instead of the deeper electropositive groove of AlgX (61). While the sequence and structural similarity between homologs with common carbohydrate-modifying functions is strong support for WssI’s role as an *O-*acetyl-esterase and/or *O*-acetyl-transferase, these models are only hypothetical, and the importance of the catalytic residues, cysteines for disulphide bond formation, and other motifs/binding sites requires biochemical verification.

### WssI^ΔN^ exhibits *O-*acetylesterase activity *in vitro*

**Figure 4:**
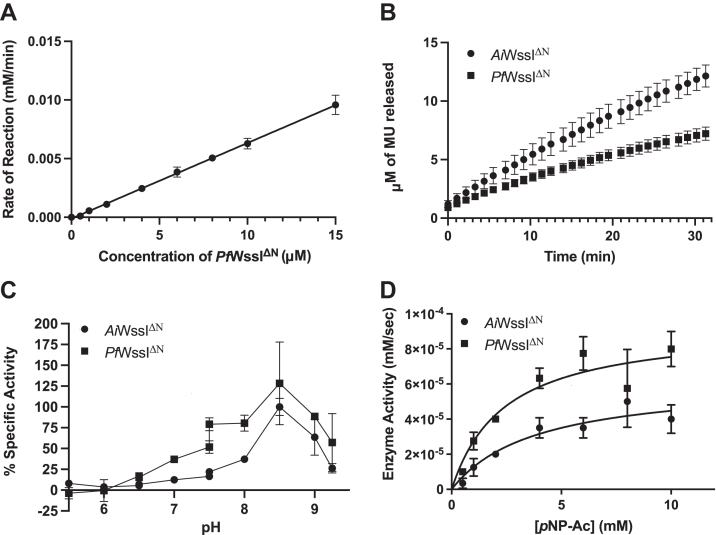
**Analysis of the enzyme activity of WssI**^**ΔN**^**.***A*, enzyme dependence of *Pf*WssI^ΔN^ reaction rates against the concentration of enzyme in the presence of 2 mM *p*NP-Ac esterase substrate tested over 30 min at 23 °C. *B*, enzyme activity of *Ai*WssI^ΔN^ and *Pf*WssI^ΔN^ against time in the presence of 0.5 mM MU-Ac esterase substrate tested at 23 °C with 2 μM of the respective enzyme. *C*, specific activity of WssI^ΔN^ proteins dependant on pH assayed for 30 min in the presence of 2 mM *p*NP-Ac substrate across 50 mM MES (pH 5.5–6.5), sodium phosphate (pH 6.5–7.5), and Tris–HCl (pH 7.5–9.25) buffers. Both WssI^ΔN^ proteins displayed the highest esterase activity at pH 8.5. *D*, representative curves for the kinetic determination of *Ai*WssI^ΔN^ (2 μM) and *Pf*WssI^ΔN^ (3 μM) *O-*acetylesterase activity with 0 to 10 mM *p*NP-Ac as substrate over 30 min at 23 °C. Kinetic parameters were determined using GraphPad nonlinear regression analysis of initial velocities. Error bars represent SD (n = 4). *Ai*WssI, WssI from *Achromobacter insuavis*; MU-Ac, 4-methylumbelliferyl acetate; *Pf*WssI, WssI from *Pseudomonas fluorescens*; *p*NP-Ac, *p*-nitrophenyl acetate.

Since WssI contains the catalytic triad of SGNH hydrolases, preliminary assays were designed to test *O-*acetylesterase activity (*i.e.*, bulk solvent serves as the acceptor instead of a carbohydrate) and determine optimal reaction parameters. Following purification of the WssI proteins lacking the N-terminal transmembrane domain (*e.g.*, *Pf*WssI^ΔN^ in Fig. S3), esterase activity was examined through a colorimetric assay with increasing concentrations of enzyme and a constant quantity of commercial acetyl donor, *p*-nitrophenyl acetate (*p*NP-Ac). The distinct trend confirmed that WssI^ΔN^ can act as an *O-*acetylesterase with this substrate, and the observed activity is enzyme dependent (Fig. 4*A*), which has been an attribute of other *O-*acetyltransferases as well (60). An alternative acetyl donor, 4-methylumbelliferyl acetate (MU-Ac) was also examined by a similar fluorescence assay, and both WssI^ΔN^ proteins again displayed increasing activity over time (Fig. 4*B*), which was also observed by *O*-acetyl-active enzymes Ape1, OatA, and PatB (65, 66, 67).

Under comparable assay conditions, the esterase activity of the WssI^ΔN^ proteins was optimized for later characterization. The pH activity profiles of *Ai*WssI^ΔN^ and *Pf*WssI^ΔN^ both demonstrated low activity between pH 5.5 and 6.5, high activity between pH 7.5 and 8.5, and then declines at higher pH values (Fig. 4*C*). All nonenzymatic hydrolysis of substrate during the pH trials and the differential absorbance of *p*NP across the pH range were accounted for with proper buffer and substrate controls. The biological relevance of the peak activity of *Ai*WssI^ΔN^ and *Pf*WssI^ΔN^ at pH 8.5 is noteworthy, as this predicts that the enzyme is likely working below its optimum *in vivo*. WssI is predicted to be a type II membrane-bound protein with its C-terminal soluble domain (containing the predicted catalytic residues) residing in the periplasm, and the pH of this cellular compartment is estimated to be between pH 5.5 and 7.5 (68). To mimic the biological system, while retaining strong activity, a pH 7 sodium phosphate buffer was chosen as the standard buffer for subsequent assay conditions.

The bell shape of the curve for the pH profile of the WssI^ΔN^ proteins is also informative, as it is similar to those noted for an *O-*acetylesterase (Ape1) and two *O-*acetyltransferases (PatB and OatA) that are active on the acetyl-carbohydrate, peptidoglycan (PG) (67, 69, 70). This finding suggests that protonation–deprotonation events are occurring since the bell shape is consistent with two ionizable groups (*i.e.*, p*K*a values around pH 6 and 9), one of which could be the imidazolium ring of histidine that has a p*K*a of 5.97. This prediction was confirmed with Ape1 and PatB when kinetic assays at each pH value were consistent with the function of the catalytic histidine within the enzymatic mechanism of these SGNH hydrolases (58, 67, 69). Thus, it is likely that the conserved catalytic histidine of WssI performs a comparable role that is dependent on its deprotonated state to activate the neighboring nucleophilic serine.

Once established, the standard assay parameters were used to ascertain that both WssI^ΔN^ proteins could be saturated at acetyl donor concentrations exceeding 5 mM *p*NP-Ac (represented in Fig. 4*D*) and further led to the determination of Michaelis–Menten kinetic parameters (Table 2). The overall kinetic trend demonstrated that both WssI^ΔN^ proteins had similar *K*_*M*_ and *k*_cat_ values, but that *Pf*WssI^ΔN^ had a higher catalytic efficiency (*k*_cat_/*K*_*M*_) than *Ai*WssI^ΔN^ (13.0 compared with 8.0 M^−1^·s^−1^, respectively) with *p*NP-Ac as the substrate. Though the WssI^ΔN^ proteins share 62% similarity at the amino acid level, there are likely to be subtle differences tuned for their respective biosynthetic systems that have yet to be revealed. While we recognize potential differences in assay preparations may occur between studies, this observation is similar to the PG *O-*acetyltransferases from *Streptococcus pneumoniae* (*Sp*OatAc) and *Staphylococcus aureus* (*Sa*OatAc) that have catalytic efficiencies (*k*_cat_/*K*_*M*_) of 289 M^−1^ s^−1^ and 52.3 M^−1^ s^−1^, respectively, with the same *p*NP-Ac acetyl donor (70). Thus, *O-*acetyltransferases acting on a similar carbohydrate can have very different efficiencies, but these results also highlight the striking discrepancies that can occur between this group of enzymes acting on different carbohydrates, as well. Indeed, the catalytic efficiency values for the WssI^ΔN^ proteins are notably smaller in magnitude compared with the PG *O-*acetyltransferases OatA (mentioned previously), PatB (*k*_cat_/*K*_*M*_ value of 797 M^−1^ s^−1^) and the SCWP *O-*acetyltransferase PatB1 (23.4 M^−1^ s^−1^) with *p*NP-Ac as the acetyl donor (67). However, the WssI^ΔN^ *k*_cat_/*K*_*M*_ values are higher than that reported for the homologous esterase/transferase AlgJ enzymes from *P. aeruginosa* (2.96 M^−1^ s^−1^) and *Pseudomonas putida* (3.39 M^−1^ s^−1^) but similar to AlgX (11.4 M^−1^ s^−1^), which are all from the alginate synthase system that more closely mimics the cellulose synthase (60, 61, 62).

**Table 2:** Kinetic parameters of the *O*-acetylesterase activity for the WssI^ΔN^ proteins

Enzyme		*K*_*M*_ (mM)	*k*_cat_ (s^−1^)	*k*_cat_/*K*_*M*_ (M^−1^·s^−1^)
*Ai*WssI^ΔN^	*p*NP-Ac	3.85 ± 1.07	3.10 × 10^−2^ ± 3.5 × 10^−3^	8.0
	MU-Ac	0.57 ± 0.24	2.58 × 10^− 3^ ± 2.8 × 10^−4^	4.5
*Pf*WssIΔN	*p*NP-Ac	2.41 ± 0.77	3.12 × 10^−2^ ± 3.4 × 10^−3^	13
	MU-Ac	0.34 ± 0.10	1.62 × 10^−3^ ± 1.6 × 10^−4^	4.8

### WssI^ΔN^ is able to bind to cellulose polymers

**Figure 5:**
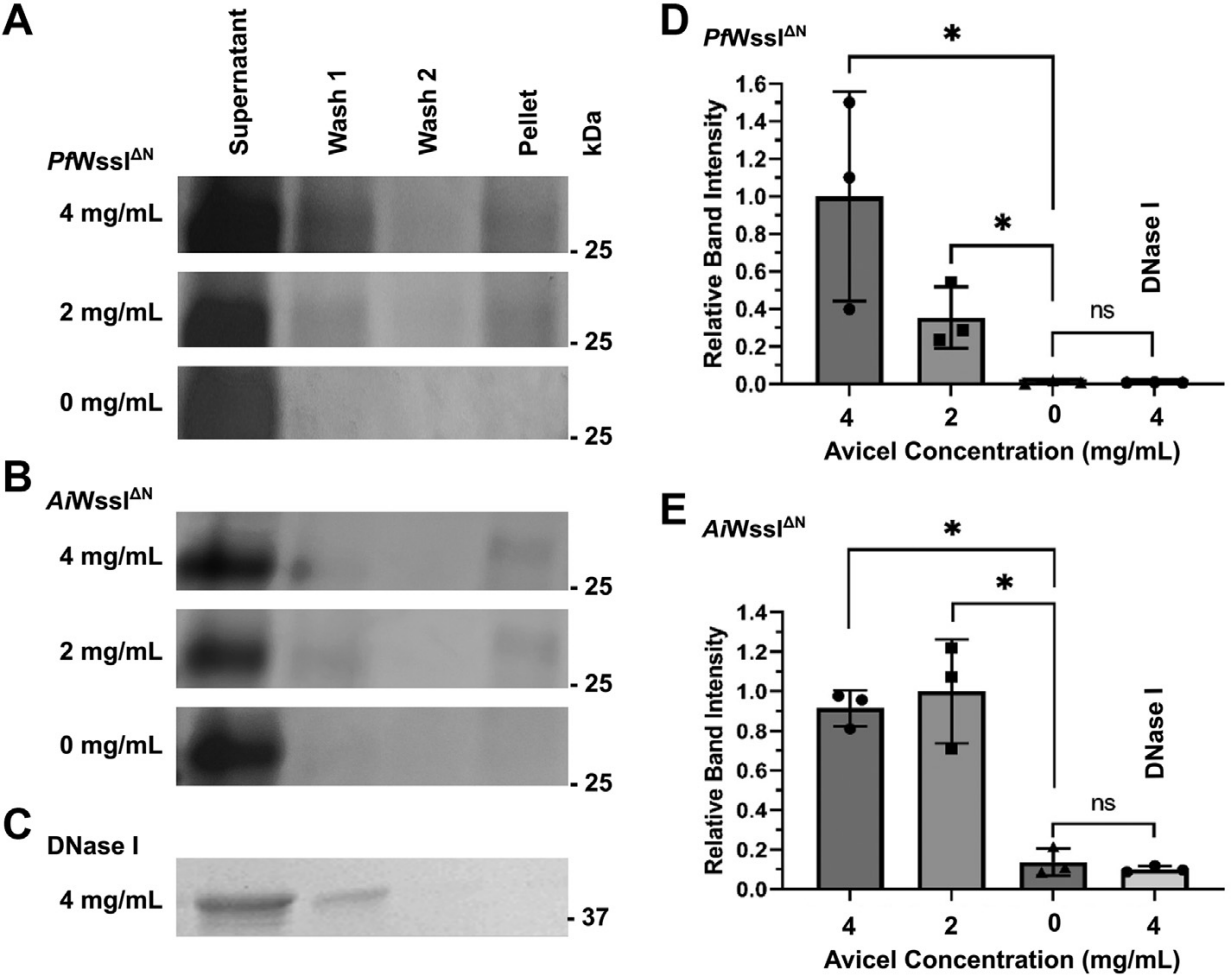
**WssI**^**ΔN**^**binding affinity for the Avicel cellulose substrate surrogate.** Representative Coomassie brilliant blue–stained SDS-polyacrylamide gels of fractions from Avicel pull-down assays with *Pf*WssI^ΔN^ (*A*), *Ai*WssI^ΔN^ (*B*), and the DNase I negative control (*C*). Assay conditions consisted of 18 h incubations at 22 °C in 50 mM sodium phosphate buffer (pH 7.0), 40 μM of the WssI^ΔN^ proteins or DNase I and Avicel concentrations as indicated. A–*C*, all tested Avicel concentrations were performed in at least triplicate, and the representative gels of these assays contain the same lane ordering from *left* to *right*: supernatant (unbound protein), washes of Avicel-protein pellet, and liberated protein from the Avicel-protein pelleted sample. *D* and *E*, graphs of increasing relative band intensities of protein liberated from Avicel-treated sample pellets at the concentrations indicated, which demonstrate significant protein binding affinity by *Pf*WssI^ΔN^ (*D*), *Ai*WssI^ΔN^ (*E*) for this surrogate substrate but not for DNase I. Relative intensities of the protein bands on the gels were analyzed using ImageJ for plotting. Statistical *t* test analysis of samples that were significant relative to the controls (*p* < 0.05) are denoted by an *asterisk*. *Ai*WssI, WssI from *Achromobacter insuavis*; *Pf*WssI, WssI from *Pseudomonas fluorescens*.

To investigate the specificity of WssI^ΔN^ for cellulose, a pull-down assay was implemented using the commercially available Avicel substrate. A finite amount of each WssI^ΔN^ enzyme was exposed to increasing concentrations of microcrystalline Avicel that was amorphous but insoluble in solution. For both enzymes, the majority of WssI^ΔN^ remained in the reaction supernatant since it was in excess, but a portion of the protein partitioned into the pellet fraction containing Avicel. This partitioned protein was not present in our controls and remained associated with the Avicel despite extensive washing (Fig. 5). Following analysis, a significant increase in both *Ai*WssI^ΔN^ and *Pf*WssI^ΔN^ concentration was noted with increasing amounts of Avicel (2 and 4 mg/ml), as detected by gel electrophoretic separation and quantification of protein from the reaction pellets relative to the no Avicel and DNase I controls (Fig. 5, *D* and *E*). These results provide initial verification that WssI^ΔN^ can bind to this polymer, despite the fact that Avicel is a substrate with a microcrystalline structure that differs from that of microbial cellulose. AlgJ from the alginate system, the closest characterized homolog of WssI known to date, only demonstrated very weak to no affinity for the enzyme’s cognate alginate substrate with mass spectrometry (MS) analyses that have far better detection capabilities than these pull-down assays (61). We recognize that direct comparison is difficult between these assays, given the difference in substrate and detection methods, but if true, a greater affinity of WssI for cellulose substrates suggests that the roles of these homologs may have diverged within their respective synthase systems.

### WssI^ΔN^ presents *O-*acetyltransferase activity *in vitro*

**Figure 6:**
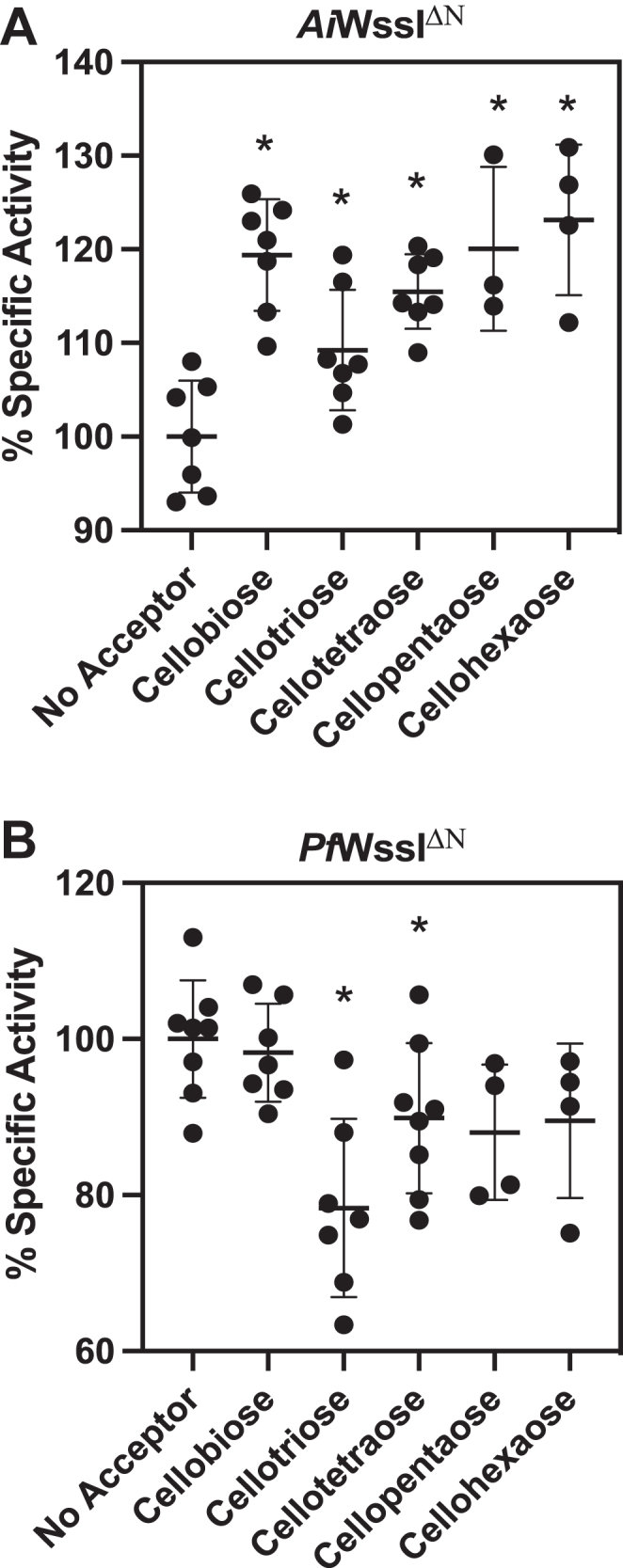
**Specific activity of *Ai*WssI**^**ΔN**^**and *Pf*WssI**^**ΔN**^**in the presence of cellulose acceptors.** Scatter plots representing specific activity trends of *Ai*WssI^ΔN^ (*A*) and *Pf*WssI^ΔN^ (*B*) against cellulose oligosaccharides (DP 2–6). Reaction rates monitoring the cleavage of *p*NP-Ac at 405 nm in the presence of cellulose acceptor were measured. The reactions were conducted in 50 mM sodium phosphate buffer (pH 7), 1 mM oligosaccharide, 3 μM (*Pf*WssI^ΔN^) or 2 μM (*Ai*WssI^ΔN^) enzyme, and 6 mM *p*NP-Ac for 30 min at 23 °C in replicates of 8. *p* < 0.05 for samples indicated by the *asterisk* from *t* test analysis; thereby, denoting statistical significance relative to the “no acceptor” controls. Significant increases in activity were demonstrated by *Ai*WssI^ΔN^ on cellobiose (*p* = 0.0017), cellotriose (*p* = 0.025), cellotetraose (*p* = 0.00028), cellopentaose (*p* = 0.018), and cellohexaose (0.0013). *Ai*WssI, WssI from *Achromobacter insuavis*; DP, degree of polymerization; *Pf*WssI, WssI from *Pseudomonas fluorescens*; *p*NP-Ac, *p*-nitrophenyl acetate.

Following on the ability of WssI^**ΔN**^ to bind to Avicel, the possibility that WssI^**ΔN**^ could function as an *O-*acetyltransferase (*i.e.*, transfer acetyl groups to an acceptor other than bulk solvent) was assessed using standard assay conditions in the presence of cello-oligosaccharide acceptors with degrees of polymerization (DP) between 2 and 6 (*i.e.*, cellobiose to cellohexaose). As noted in Figure 6, the specific activity of *Pf*WssI^ΔN^ did not increase above the basal hydrolysis rate (*i.e.*, no celloacceptor present) when incubated with the cello-oligosaccharides. In contrast, *Ai*WssI^ΔN^ demonstrated significant increases in activity in the presence of acceptors with DP ≥4. Specifically, *Ai*WssI^ΔN^ activity had 28 and 31% increases between the reaction without celloacceptors and those containing cellopentaose and cellohexaose, respectively. The specificity of *Ai*WssI^ΔN^ for cello-oligosaccharides was confirmed when reactions were repeated in the presence of chito-oligosaccharides (DP 2–6), and there was no significant increase in activity above the basal rate of hydrolysis (*i.e.*, in the absence of chitoacceptors) (Fig. S4). Similar to the discrepancy in kinetic efficiency between *Pf*WssI^ΔN^ and *Ai*WssI^ΔN^ noted previously, the esterase effects in the presence of celloacceptors highlight the value in characterizing the two WssI enzymes from unique organismal backgrounds and further demonstrate the subtle differences tuned for the respective biosynthetic systems.

**Figure 7:**
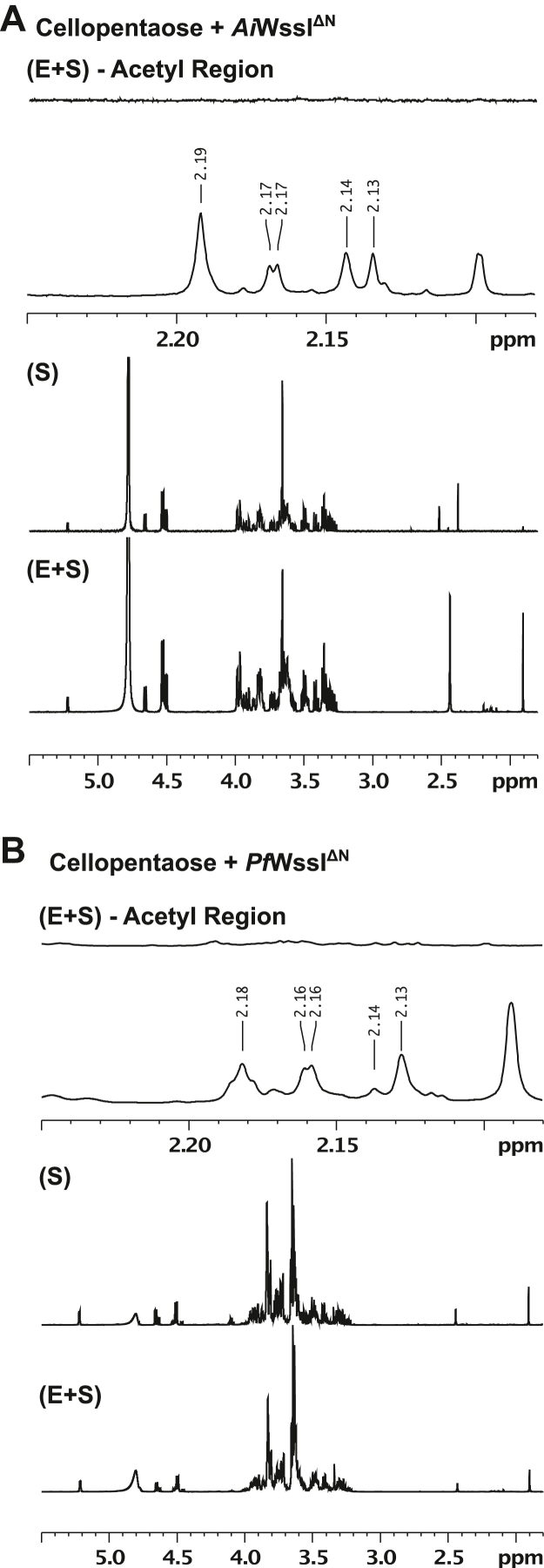
**^1^H NMR spectra of acetylated cellopentaose reaction products by *Ai*WssI^ΔN^ and *Pf*WssI^ΔN^.** Assays were conducted in 50 mM sodium phosphate buffer (pH 7) containing 3 mM cellopentaose, 20 μM WssI^ΔN^, and 2 mM MU-Ac as the acetyl donor over 2 h at 22 °C. For both (*A*) *Ai*WssI^ΔN^ and (*B*) *Pf*WssI^ΔN^, the spectra are arranged on top of each other for reference with the ([E + S] – acetyl region) spectra depicting a magnified portion of the WssI^ΔN^ enzyme–treated sample showing the multiple acetyl proton peaks between 2.25 and 2.08 ppm, the (E + S) spectra depicting the full ^1^H NMR spectra of the enzyme-treated sample, and the (S) spectra of the enzyme-free sample (*i.e.*, negative control) lacking acetyl proton peaks. *Ai*WssI, WssI from *Achromobacter insuavis*; MU-Ac, 4-methylumbelliferyl acetate; *Pf*WssI, WssI from *Pseudomonas fluorescens*.

To expand on these results, NMR spectroscopy was performed to verify acetylation of the cellopentaose products and uncover the regiospecificity of the acetyl modification on the carbohydrate monomers. Acetylation of cellopentaose was noted only in the WssI^ΔN^-treated samples (Figs. 7 and S5), but at multiple positions, which corresponds to one position (*e.g.*, C2) on one polymer and in another position (*e.g.*, C3 or C6) on another cellopentaose. Thus, the product proton peaks for acetylation are split across these modification positions resulting in lower signal intensity that make it difficult to identify specific acetyl positions and precise anomeric carbon proton peaks (as noted in the DOSY experiments where proton peaks are along the same translational shift but not separated enough for specific correlation; Fig. S5). Multiple trials to improve acetyl-product detection were attempted (*i.e.*, increasing substrate and enzyme concentrations, combined with extensive incubations and subsequent cellulase treatment to render the product into monomeric components), but the high concentration of product needed for analysis and the lack of complete conversion of substrate by WssI^ΔN^ preclude regiospecificity identification by this method under these conditions. However, the identification of multiple acetylation positions is consistent with the work of Spiers *et al.*, which also found acetylation at multiple positions (*i.e.*, on the 2, 3, and 6 carbons) in cellulose isolated from *P. fluorescens.* Furthermore, integration of the area of all the acetyl peaks compared with the anomeric proton peaks suggests that the amount of acetylated product (assuming it is all monoacetylated as noted by MS below) was approximately 9.4%, which compares favorably to the 14% *O-*acetylation level found by Spiers *et al.*

**Figure 8:**
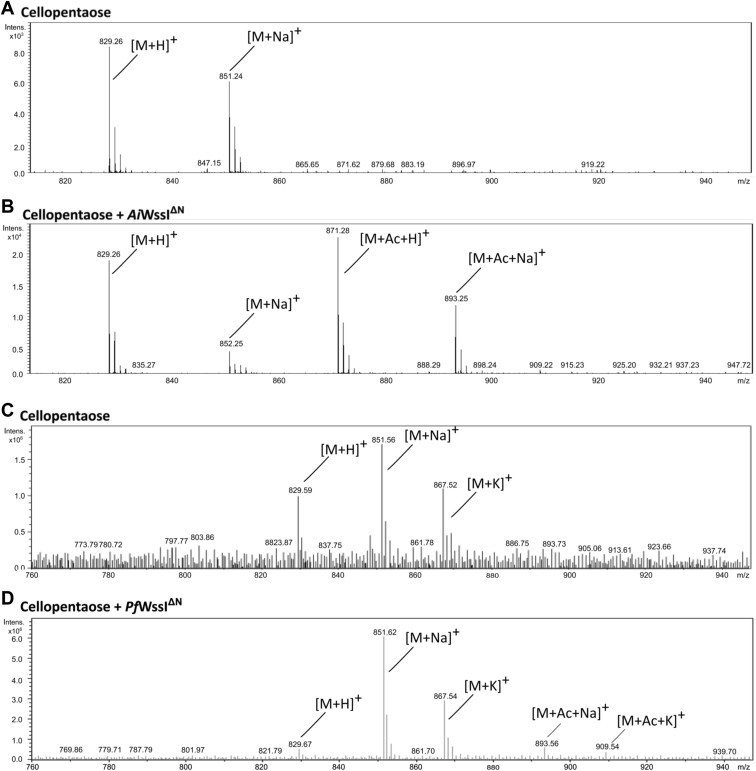
**Electrospray ionization–MS analysis of acetylated cellopentaose reaction products by *Ai*WssI**^**ΔN**^**and *Pf*WssI**^**ΔN**^**.** Assays were conducted in 50 mM sodium phosphate buffer (pH 7) containing 1 mM cellopentaose, 3 μM (*Pf*WssI^ΔN^) or 2 μM (*Ai*WssI^ΔN^) enzyme, and 6 mM *p*NP-Ac as the acetyl donor over 30 min at 23 °C. All cellulose oligomer samples were purified from reaction components by solid-phase extraction prior to analysis. The (*A*) and (*C*) mass spectra were obtained for the cellulose oligosaccharides as a baseline control; (*B*) and (*D*) mass spectra were obtained for the enzyme-treated cellopentaose reactions. M denotes the molecular mass of cellopentaose (828 Da) in the respective spectra, whereas H^+^, Na^+^, K^+^, and Ac denote *m/z* increases consistent with the addition of a hydrogen (1 Da), sodium (23 Da), potassium (39 Da), and/or an acetyl group (42 Da) to cellopentaose. Acetyl transfer was observed by both *Ai*WssI^ΔN^ and *Pf*WssI^ΔN^ to this oligosaccharide. All spectra were obtained in positive polarity mode. *Ai*WssI, WssI from *Achromobacter insuavis*; *Pf*WssI, WssI from *Pseudomonas fluorescens*; *p*NP-Ac, *p*-nitrophenyl acetate.

With knowledge that WssI^**ΔN**^ interacts with multiple forms of cellulose (*i.e.*, Avicel and cello-oligomers) and that reaction rates are influenced by polymer length, the reactions containing cellopentaose were assessed after solid-phase extraction of the oligosaccharides to further characterize acetyltransfer events. MS analysis of the control reactions (Fig. 8, *A* and *C*) identified expected adducts of cellopentaose (*m/z* species for hydrogen, sodium, and potassium adducts of 829, 851, and 867, respectively). Both *Ai*WssI^ΔN^- and *Pf*WssI^ΔN^-treated samples not only possessed the same substrate adducts but also contained new mass species with the addition of 42 Da, which is consistent with the mass of a single acetyl group, to the adducts of cellopentaose (Fig. 8, *B* and *D*), which confirmed that acetyltransfer had occurred. While complete conversion could not be achieved with these substrates, *Ai*WssI^ΔN^ displayed more relative product conversion compared with the starting material than that achieved with *Pf*WssI^ΔN^ despite there being less *Ai*WssI^ΔN^ (*e.g.*, 2 μM compared with 3 μM, respectively) under otherwise common assay parameters. The sensitivity of MS detection is underscored by the fact that the specific activity values for *Pf*WssI^ΔN^ using the acetylesterase assays (noted previously) did not give an indication of transferase activity onto cellulose polymers of any length, yet with MS we detected transfer onto cellopentaose.

**Figure 9:**
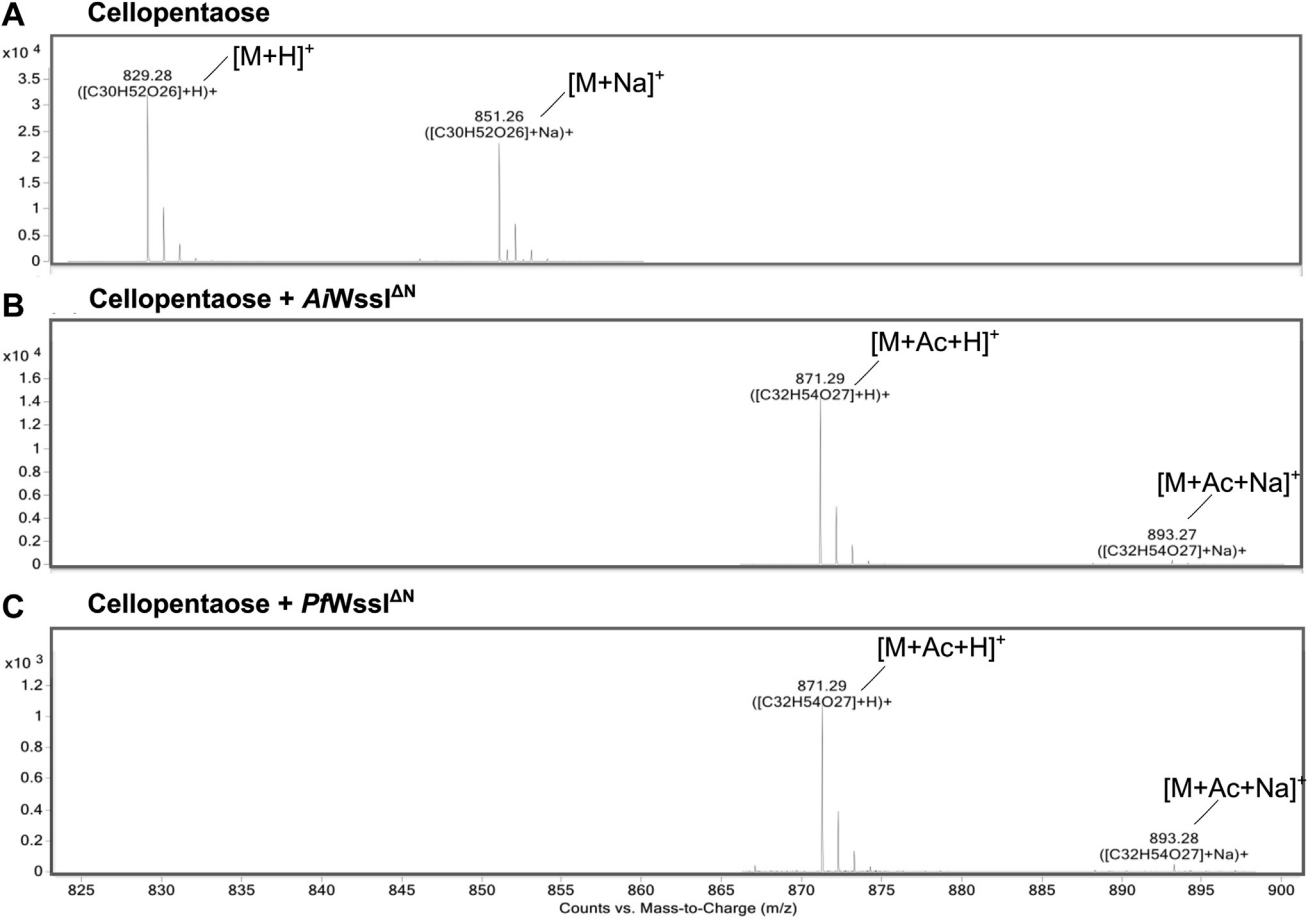
**LC–MS analysis of reaction products by *Ai*WssI**^**ΔN**^**and *Pf*WssI**^**ΔN**^**with acetyl-CoA and cellopentaose.** Reactions were conducted in 50 mM sodium phosphate buffer (pH 7), 1 mM cellopentaose, 3 μM (*Pf*WssI^ΔN^) or 2 μM (*Ai*WssI^ΔN^) enzyme, and 4 mM acetyl-CoA as the acetyl donor. The (*A*) mass spectra were obtained for cellopentaose as a baseline control; (*B*) and (*C*) mass spectra were obtained from the *Ai*WssI^ΔN^ and *Pf*WssI^ΔN^ reactions following 24 h enzyme treatment at 23 °C, respectively. M denotes the mass of cellopentaose (828 Da), whereas H^+^, Na^+^, and Ac denote that *m/z* increases are consistent with the addition of a hydrogen (1 Da), sodium (23 Da), and/or an acetyl group (42 Da) to cellopentaose. All spectra were obtained by LC–electrospray ionization mass spectrometry in the positive polarity mode following separation with C18 reverse-phase chromatography. *Ai*WssI, WssI from *Achromobacter insuavis*; *Pf*WssI, WssI from *Pseudomonas fluorescens.*

Transfer conformation analysis *via* MS was also used to assess acetyl-CoA as an alternative donor substrate for acetyltransfer. While acetyl-CoA is unlikely to be present in the periplasm where WssI is predicted to function, it is at least a common metabolite associated with carbohydrates and lipids within bacterial cells and has also been noted to be a substrate for membrane-bound *O*-acyltransferase (MBOAT) proteins that are predicted to perform the immediately preceding step to WssI in acyltransfer onto polysaccharides outside the cell (71, 72, 73, 74). A standard activity assay with acetyl-CoA as the substrate was prepared, and the appropriate controls were separated and assessed using LC–MS analysis. The control reaction contained the appropriate masses of a cellopentaose with hydrogen and sodium adducts (*m/z* species 829.28 and 851.56, respectively) (Fig. 9*A*) but not acetylated polymer. In contrast, *Ai*WssI^ΔN^- and *Pf*WssI^ΔN^-containing reactions possessed the cellopentaose adducts with an additional 42 Da mass that was again consistent with the addition of a single acetyl group onto the polymer (see *insets* for Fig. 9, *B* and *C*). Similar to the aforementioned *p*NP-Ac acetyl donor MS analyses, a higher abundance of products for the *Ai*WssI^ΔN^ containing reaction was noted when compared with *Pf*WssI^ΔN^ despite there being less *Ai*WssI^ΔN^ under otherwise common assay parameters. These results support that *Ai*WssI^ΔN^ has the higher turnover rate with this acceptor, which is also in line with the increased *Ai*WssI^ΔN^ acetylesterase activity in the presence of cellopentaose and cellohexaose. Intriguingly, results from similar experiments with AlgJ from the alginate system could only demonstrate weak binding to the enzyme’s cognate alginate substrate (*i.e.*, through MS analyses) but not acetyltransfer onto carbohydrate oligomers (61). Regardless of enzyme variant, the *O-*acetyltransferase activity observed with the surrogate donor substrates (*p*NP-Ac, MU-Ac, and acetyl-CoA) is only likely to increase with the native biological substrates and in the presence of the other WssFGH proteins that are part of the cellulose acetylation machinery. Indeed, the leading model postulates that WssI relays the acetyl group from WssH (MBOAT protein) to WssF for acetylation of glucose units as the cellulose polymer passes through the periplasmic layer prior to export (50). It should be noted that recent crystal structures of MBOAT proteins have displayed a common funnel shape that protrudes into the cytoplasmic membrane with a conserved catalytic histidine (74). In these systems, the conserved histidine is proposed to play a role in direct activation of a periplasmic substrate so that it can perform nucleophilic attack on acyl or alanyl groups attached through thioesters, like that of acetyl-CoA, that are presented from the cytoplasmic side of the membrane. However, recent work with *Sa*OatA suggests that a conserved tyrosine can also fulfill this role (74). Given that WssI^**ΔN**^ was able to accept acetyl groups from both hydroxyl- and sulfhydryl-based donors (*i.e.*, *p*NP-Ac and acetyl-CoA), it remains unclear what amino acid residues (*e.g.*, Glu, Asp, Cys, Tyr, etc.) or accessory components are used by WssH to present the acetyl groups to the periplasmic face of the bacterial inner membrane for recognition by WssI and/or WssF to then subsequently transfer onto cellulose polymers.

### Inhibitor and activity profiling of WssI^ΔN^

Investigation into the effects of the cysteines (C102 and C160), predicted to form a disulfide bond in our structural model, were probed by performing purifications and assays in the presence and absence of DTT reducing agent. Interestingly, purifications lacking DTT had approximately 90% lower activity (*i.e.*, 11.9 ± 1.12% and 7.1 ± 3.74% residual activity for *Ai*WssI^ΔN^ and *Pf*WssI^ΔN^, respectively, compared with in the presence of DTT). These findings suggest that the two cysteine residues may be required in a reduced form in order for catalysis by WssI to occur, but the possibility that the oxidized/reduced states of other residues in the protein may also be contributing to this effect cannot be overlooked. However, there was no noticeable difference in the secondary structure of the WssI^ΔN^ proteins in the presence of DTT when analyzed by CD spectroscopy or using tryptophan fluorescence spectroscopy (Fig. S2), so the effects (if present) are likely localized to the binding site region near the disulfide bond. In work with AlgX from *P. aeruginosa*, comparable Cys residues in the α/β cap region above the active site exist as a disulfide bond in the active state (61), so it is plausible that the oxidized/reduced state of these residues in these *O-*acetyltransferases may be influencing proper positioning of neighboring residues that are involved in binding and catalysis. While disulfide bonds can also be found in some of the other related SGNH enzymes (*e.g.*, PatB1 and XOAT1) (60, 62, 64), none of these disulfides are conserved in location, and other homologs have no disulfides at all, so the influence of disulfides on active-site residues is likely to be enzyme specific.

A targeted inhibitor profile was also performed on the WssI^ΔN^ proteins in an effort to use these molecular tools to probe the character of the active site and provide insight into the catalytic mechanism. Commercially available serine protease/esterase inhibitors, like PMSF and methanesulfonyl fluoride (MSF), have been used in the literature to abrogate the function of SGNH hydrolases, by irreversibly binding to the catalytic serine and preventing turnover of the enzyme (75). Although WssI is predicted to have functionality using the catalytic triad of SGNH hydrolases, extensive incubation with PMSF or MSF (*i.e.*, possessing a methyl group instead of the bulkier phenyl group of PMSF) did not lead to significant reduction in activity (Table 3). Unfortunately, higher concentrations of both PMSF and MSF resulted in precipitation; thereby, precluding the full extent of inhibitor effects on the WssI^ΔN^ proteins. However, a similar lack of inhibition by these chemicals has also been noted for other carbohydrate *O-*acetyl-transferases and *O*-acetyl-esterases (*e.g.*, OatA, PatB, and Ape1) of the SGNH family (59, 60, 69). These combined results imply that PMSF, and in some cases MSF, is not a suitable *in vitro* inhibitor for these unconventional serine-active variations of the Ser-His-Asp catalytic residues (57, 59, 64).

**Table 3:** Effect of reagents on WssI^ΔN^ activity

Reagent	Residual activity (%)^a^	
	*Pf*WssI^ΔN^	*Ai*WssI^Δ*N*^
1 mM PMSF	103.2 ± 4.2	106.3 ± 6.8
5 mM MSF	112.1 ± 4.4	95.0 ± 6.2
5 mM EDTA	88.3 ± 1.6	89.3 ± 1.5
DTT absent^b^	7.1 ± 3.74	11.9 ± 1.1
0.1 mM E-64	107.8 ± 10.8	130.8 ± 9.1
20 μM Pepstatin A	98.9 ± 6.0	140.7 ± 6.5

a All reactions were performed in at least quadruplicate in 50 mM sodium phosphate buffer (pH 7) and initiated by the addition of 6 mM *p*NP-Ac following incubation of WssI^ΔN^ with a listed effector for 1 h.

b These reactions were performed as the others but in the absence of 1 mM DTT reagent.

To expand the inhibitor profile and explore the incongruous possibility that WssI was working through a nonserine catalytic mechanism, additional inhibitors were also assayed. Pepstatin A specifically inhibits catalytic aspartate residues and E-64 targets cysteines, but neither of these inhibitors eliminated the activity of WssI^**ΔN**^ at supplier-recommended concentrations (Table 3). The WssI^**ΔN**^ proteins were also preincubated with the EDTA metal chelator prior to assays, but the enzymes retained approximately 90% activity; thereby, indicating that WssI is unlikely to have metalloenzyme catalytic properties either. In fact, the minimal reduction in activity with regard to WssI may instead be due to the loss of metals involved in protein stability, since protein degradation was observed during incubation with high concentrations of EDTA (*i.e.*, 5 mM) for prolonged periods.

**Figure 10:**
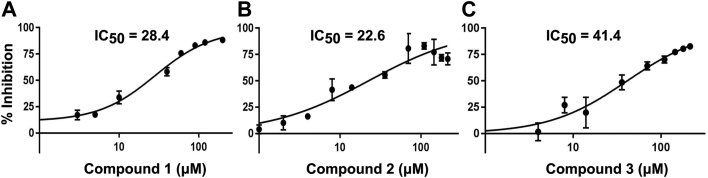
**Dose–response curves of *Ai*WssI**^**ΔN**^**and hit compounds.** Dose–response curves of percent inhibition of *Ai*WssI^ΔN^ against compound 1 (*A*), compound 2 (*B*), and compound 3 (*C*) with reported IC_50_ values of 28.4, 22.6, and 41.4 μM, respectively. MU-Ac (0.5 mM) was the substrate for the assay, and error bars denote standard deviation (n ≥ 3). *Ai*WssI, WssI from *Achromobacter insuavis*; MU-Ac, 4-methylumbelliferyl acetate.

Since commercial-based inhibitors of activity proved elusive, HTS of novel compound libraries was employed instead to uncover inhibitors of the acetylesterase reaction of WssI. Using the MU-Ac fluorescence esterase assay adapted for HTS, *Zʹ* values for *Ai*WssI^ΔN^ were always between 0.5 and 0.7 for each independent enzyme preparation and HTS assay (*i.e.*, across greater than 60 test plates); thereby, providing a quality indicator that there was an appropriate window of responsivity to detect inhibition well beyond typical standard error (see Fig. S6 as a data example). The primary screen of *Ai*WssI^ΔN^ involved testing against 18,425 compounds from the Chembridge DIVERset library collection, along with 135 other compounds that were included based on their success against other SGNH enzymes. Following initial (singly) and confirmatory screening (triplicates), there were 50 identified hits with at least two of the three replicates with Z scores <−3 (0.27% hit rate). These 50 compounds were analyzed against Pan-Assay INterference compound (PAINS) indicators using Tables 11 and 12 from the study of Baell and Holloway (76) for substructures that may cause assay interference and false-positive hits. Thirty-eight of these compounds were assessed to have minimal PAINS and had substructures that were amenable to downstream medicinal chemistry manipulations. Dose–response curves for each of these compounds against *Ai*WssI^ΔN^ were conducted to determine individual IC_50_ values. After careful consideration, a list of eight compounds were chosen for further analysis (Table 4) since these compounds were able to saturate *Ai*WssI^ΔN^ (*i.e.*, approached 100% inhibition) and had IC_50_ values in the micromolar range for the esterase assay using MU-Ac as the substrate. Each of these compound hits were purchased and further verified in our MU-Ac in-house standard assay (approximately six times more volume than the HTS assay), and five of the compounds gave inconsistent results either based on compound precipitation over time or inability to reach 100% inhibition in both versions of the assay. The remaining three compounds with promise exhibited IC_50_ values in the low micromolar range across both versions of the trials and had inhibition constant (*K*_*i*_) values of 15.1, 12.0, and 22.1 μM for compounds 1, 2, and 3, respectively (Table 4; Fig. 10).

**Table 4:** Dose–response results for active compounds against *Ai*WssI^ΔN^

ID	Compound name	Structure	*M*_*w*_ (g mol^−1^)	HTS MU-Ac	Second MU-Ac	
				IC_50_ (μM)^a^	IC_50_ (μM)^b^	*K*_*i*_ (μM)^b^
1	(3Z)-2-Amino-3-[(4-hydroxyphenyl)-methylidene]prop-1-ene-1,1,3-tricarbonitrile^c^		236.2	10.1	28.4	15.1
2	Ethyl *N*-[(2E)-2-cyano-2-{[4-(dimethylamino)-phenyl]methylidene}acetyl]carbamate		287.3	60.2	22.6	12.0
3	(2Z)-3-(1-Methyl-1H-pyrrol-2-yl)-2-(4-methyl-5-phenyl-4H-1,2,4-triazol-3-yl)-prop-2-enenitrile		289.3	85.3	41.4	22.1
4	*N*-[6-(4-Methylpiperazin-1-yl)pyridin-3-yl]-2-(phenylamino)thieno[2,3-days][1,3]thiazole-5-carboxamide		450.6	55.3		
5	(4E)-4-[3-(Furan-2-yl)prop-2-en-1-ylidene]-1,2,3,4-tetrahydroisoquino-line-1,3-dione		256.3	51.9		
6	1-{2-Oxo-3-[(2E)-1,3,3-trimethyl-2,3-dihydro-1H-indol-2-ylidene]propyl}-5-(pyrrolidine-1-sulfonyl)-1,2-dihydropyridin-2-one		441.6	71.9		
7	*N*-{4-[(4-Nitrophenyl)sulfamoyl]phenyl}acetamide		335.3	100.0		
8	4-[(2E)-2-{[4-Methyl-2-(piperidin-1-yl)-1,3-thiazol-5-yl]methylidene}hydrazin-1-yl]benzoic acid		344.4	90.3		

a Using MU-Ac as the esterase substrate in the HTS fluorescence assays.

b Using MU-Ac as the esterase substrate in the follow-up standard assay.

c Common compound name is Tyrphostin AG 112.

Of the three promising compounds, compound 1 ((3Z)-2-amino-3-[(4-hydroxyphenyl)-methylidene]prop-1-ene-1,1,3-tricarbonitrile) is commercially known as Tyrphostin AG 112. This compound works on tyrosine kinases through competitive inhibition of phosphoryl group transfer and therefore has gained recognition for its antiproliferative activity with regard to proteins like epidermal growth factor receptors (77, 78). While typically used to inhibit tyrosine-active enzymes, tyrphostins have also been shown to inhibit the serine/threonine phosphatase calcineurin in the micromolar range (IC_50_ of 30 μM for tyrphostin AG112 with *p*-nitrophenyl phosphate as a substrate); thereby, suggesting a possible broader role for these inhibitors (78). The connection to WssI and its low micromolar inhibition with MU-Ac similarly involve inhibition of the cleavage of serine-linked ester bonds; however, for this enzyme, hydrolysis of acetate from an acetyl donor would be prevented instead of a phosphoryl group.

## Conclusions

In this study, we have demonstrated that WssI^ΔN^ exhibits acetylesterase and acetyltransferase activity *in vitro*. This is the first biochemical characterization of a protein from the Wss protein system, which corroborates the predicted role of the WssFGHI proteins to be responsible for acetylation of bacterial cellulose, as previously identified by Spiers e*t al.* through phenotypic mutants (18, 46). We outlined optimal enzyme conditions for functional assays, length-dependent substrate specificity with cellulose oligomers, and acetyltransfer by NMR and MS analysis for WssI^ΔN^ from two organismal backgrounds, *P. fluorescens* and *A. insuavis*. Through these combined results, we have demonstrated that WssI^ΔN^ first participates in the cleavage of an acetyl donor substrate, followed by transfer onto either bulk solvent (esterase reaction) or onto a cellulose acceptor (transferase reaction). Furthermore, the WssI^ΔN^ proteins were able to demonstrate this activity with different substrate donor mimetics (*e.g.*, *p*NP-Ac, MUB-Ac, and acetyl-CoA) and acceptors (Avicel and short cello-oligomers).

In the alginate system, AlgJ is proposed to be an intermediary protein in the acetylation pathway and a second SGNH protein, AlgX, is the terminal *O-*acetyltransferase (61), which differs from the PG and SCWP systems that lack an intermediary protein (59, 60). The *wss* system appears to be analogous to the alginate system in that it also has an AlgX homolog (WssF), noted most recently in *Bordetella avium*, but this protein has yet to be biochemically characterized (13). In *K. xylinus*, the *bcsII* operon is also proposed to have two SGNH proteins (*bcsX* and the C-terminal domain of *bcsZ*), but their activities on acylated cellulose substrates remain unknown (12). Based on the similarity to the alginate system, the cellulose acetylation machinery would also posit WssI as the intermediary protein and WssF as the terminal *O-*acetyltransferase. However, our *in vitro* assays with WssI^ΔN^ demonstrating greater overall catalytic efficiency and successful acetyltransfer onto cellulose suggest that the role of WssI may be different than that of AlgJ. It is plausible that there may have been an evolutionary adaptation to use two terminal *O-*acetyltransferases to match an increasing demand of synthesis, acetylation, and export of the cellulose exopolysaccharides, which are transiently passing through the periplasm of these bacteria under conditions requiring high biofilm production. Indeed, the extent of O-acetylation of comparable PG and alginate polymers varies between 20 to 70% and 4 to 57%, respectively, depending on species, strain, and culture conditions (79, 80), so it is likely that the bacterial cellulose system is also finely tuned to fit unique requirements. In the alginate system, there is also evidence based on protein interaction work that all AlgIFJ and X form a complex that work together for proper acetylation of this polymer (25, 46, 81). However, whether WssF is truly a second *O-*acetyltransferase in the cellulose synthase complex and if it works separately or in conjunction with WssI still needs to be investigated along with the protein stoichiometry of the remaining Wss proteins. Regardless, it is likely that the activity of WssI within the cellulose synthase background is higher, given that it would have its proper complement of protein partners, access to the true biological substrates (as opposed to the substrate mimetics employed in our assays), and arranged in such a way as to promote acetyltransfer as opposed to acetylesterase activity.

As a cellulose *O-*acetyltransferase, WssI likely participates directly in the acetylation of microbial cellulose found in the biofilms of *P. fluorescens* and *A. insuavis* and therefore contributes to the unique chemical and physiological properties of these biofilms that are adapted to certain environmental niches. While our initial targeted work with common inhibitors proved fruitless, the subsequent extensive HTS trials yielded the three first known inhibitors of cellulose acetylation, complete with IC_50_ values in the micromolar range that is desirable as a starting point for further chemical augmentation/engineering. By exploiting WssI and the associated acetylated-cellulose biosynthetic proteins as druggable targets, the potential for less adherent and/or eradication of biofilms associated with persistent diseases can advance treatment options and aid in mitigating the economic loss caused by these organisms (33, 34, 35, 36, 37, 38, 39, 40). In a contrasting avenue, our knowledge of WssI also lays the groundwork for the possible promotion of biofilms formed by these bacteria in situations that are beneficial to certain crop cultivars (*i.e.*, in tomato, wheat, and tobacco) (41, 42). While these novel findings represent an important area of study to understand robust biofilm formation by these model organisms, they also extend to the growing list of organisms that possess homologs of the *wss* gene cluster and offer a juxtaposition to bacteria that instead use the recently identified phosphoethanolamine modification of cellulose (15, 20, 21, 22). Finally, this work not only expands our knowledge of the O-acetylation of polysaccharides in bacterial biofilms by demonstrating both parallels to the prototypical alginate system but also areas that are finely tuned to these unique polymers that are likely to have broad implications for the colonization and persistence of particular niches, like the lungs of cystic fibrosis patients that are colonized by both *Pseudomonas* and *Achromobacter* species (31, 37).

## Experimental procedures

### Cloning and bioinformatic analysis

The plasmid containing the *A. insuavis wssI* sequence was purchased from Genscript biotech in a pET24 plasmid background and designed so that *wssI* is in frame with a coding sequence for a six-histidine residue tag located on the carboxyl terminus (p*Ai*WssI^ΔN^-His_6_) (Table S1). The p*Ai*WssI-His_6_ plasmid was also designed so that *wssI* consists of the gene region lacking the N-terminal transmembrane section (amino acids 1–94) that was noted by the PSort server (82), so only the predicted soluble domain of WssI was produced (285 amino acids in length; termed *Ai*WssI^ΔN^). Similarly, the *wssI* gene from *P. fluorescens* (*Pf*WssI) was successfully cloned into a pET28 plasmid vector with a C-terminal His_6_-tag (p*Pf*WssI^ΔN^-His_6_) containing the comparable soluble domain from this organism (282 amino acids in length; missing amino acids 1–91 and termed *Pf*WssI^ΔN^) (Table S1). The WssI^ΔN^ proteins have calculated molecular weights of 31.48 kDa (*Pf*WssI^ΔN^) and 31.68 kDa (*Ai*WssI^ΔN^).

Bioinformatic analyses involving the full protein sequences and the soluble truncated derivatives (*i.e.*, *Pf*WssI^ΔN^ and *Ai*WssI^ΔN^) were conducted using ProtParam for general protein characteristics (https://web.expasy.org/protparam/), Phyre2 and AlphaFold2 for homology modeling and secondary structure prediction (83, 84, 85), and Clustal Omega for sequence alignments as well as identity determinations (https://www.ebi.ac.uk/Tools/msa/clustalo/) (86). The TMHHM server was used to obtain a topology model (http://www.cbs.dtu.dk/services/TMHMM/) (87) that, along with results from the AlphaFold2 data, was rendered in two-dimensional format with TopDraw (88). The molecular models, structural superpositions (using the cealign plugin), and electrostatic surface prediction (using the ABPS electrostatics plugin) were rendered in PyMOL (The PyMol Molecular Graphics System, version 2.4.2; Schrödinger, LLC).

### Expression conditions

All plasmids were propagated in *E. coli* TOP10 cells and purified where necessary using a GeneJET Plasmid miniprep kit (Thermo Scientific). The plasmids were then transformed into, screened, and maintained in chemically competent *E. coli* BL21 (Rosetta Codon Plus) cells (Table S1). Successful transformants were used for growth cultures, which were supplemented with kanamycin and chloramphenicol (50 and 34 μg/ml, respectively) and grown for 16 to 18 h at 37 °C with agitation. For large-scale protein expression, 1 l of enriched broth growth media (32 g tryptone, 20 g yeast extract, and 10 g NaCl, supplemented with 50 μg/ml of kanamycin and 34 μg/ml of chloramphenicol) was inoculated and incubated at 37 °C with agitation until an absorbance at 600 nm of 0.6 was reached. Expression of protein from p*Ai*WssI^ΔN^-His_6_ or p*Pf*WssI^ΔN^-His_6_ was induced by the addition of 1 mM isopropyl β-d-1-thiogalactopyranoside, and the culture was grown for an additional 16 to 18 h at 17 °C before harvesting.

### Purification of WssI

Cell pellets from expression trials were resuspended in lysis buffer (50 mM Tris [pH 7.5], 500 mM NaCl, 1 mM DTT, and 2% [v/v] glycerol) that also contained RNaseA (final concentration of 16 μg/ml), DNase I (final concentration of 8 μg/ml), 20% (w/v) lysozyme, and 0.1% (v/v) Triton X-100. The bacterial suspension was incubated on a rocking platform at 4 °C for 30 min and mechanically lysed by passing the sample twice through a constant cell disruption system (Constant Systems Ltd) at 17 kpsi. Unwanted large cellular debris was removed through centrifugation (28,000*g* for 45 min at 4 °C),and the clarified soluble cellular lysate was retained. The clarified lysate was then adjusted to contain 20 mM imidazole to prevent nonspecific binding during affinity chromatography. Using an AKTA Start Fast Protein LC system, the lysate was applied to a 5 ml HisTrap FF column (GE Healthcare Life Sciences) and washed with 3 column volumes (CVs) of lysis buffer containing 20 mM imidazole. An additional wash step was done by applying 4 CVs of 90% lysis buffer and 10% elution buffer (consisting of lysis buffer with 500 mM imidazole). Elution was accomplished with a linear gradient that transitioned into 100% elution buffer across 10 CVs, with both WssI^ΔN^ proteins eluting at approximately 175 mM imidazole concentration. Fractions containing WssI (determined by SDS-PAGE as samples containing bands of the correct predicted molecular weight) were pooled and dialyzed (molecular weight cutoff = 14,000 Da) against 50 mM Tris (pH 7.5), 1 mM DTT, and 2% (v/v) glycerol at 4 °C with gentle stirring for approximately 1 h, after which the buffer was replaced and allowed to dialyze for another 16 to 18 h.

Subsequent purification was performed by anion exchange chromatography with a 5 ml HiTrap Q FF column (GE Healthcare Life Sciences). Purification was conducted over 6 CVs of a linear gradient from 0 to 500 mM NaCl in ion exchange buffer (50 mM Tris [pH 7.5], 1 mM DTT, and 2% [v/v] glycerol). Purified WssI was dialyzed (molecular weight cutoff = 14,000 Da) against assay buffer (50 mM sodium phosphate [pH 7.0], 150 mM NaCl, 2% [v/v] glycerol, and 1 mM DTT) prior to use.

The concentration of the protein was measured using the Gen5 program (settings include absorbance at 280 nm detection and molar extinction coefficients 33,585 M^−1^ cm^−1^ and 50,085 M^−1^ cm^−1^ for *Pf*WssI^ΔN^ and *Ai*WssI^ΔN^, respectively) on a Cytation 5 microplate reader (VWR), corrected against the assay buffer background. A total protein yield from 1 l of culture typically resulted in 0.8 and 0.125 mg/ml after the HisTrap FF and anion exchange chromatography columns, respectively, for *Ai*WssI^ΔN^. Purification of *Pf*WssI^ΔN^ yielded slightly more protein, with 1.5 and 0.25 mg/ml obtained after HisTrap FF and anion exchange purification, respectively. WssI^ΔN^ was either used directly or stored in assay buffer with 10% (v/v) glycerol at −20 °C until needed.

### Measurement of enzymatic activity

The enzymatic rate of the WssI^ΔN^ proteins was measured using chromogenic assays in a 50 mM sodium phosphate-buffered (pH 7) solution, with WssI^ΔN^ (3 μM unless specified otherwise) and were initiated by the addition of *p*NP-Ac to a final concentration of 6 mM (dissolved in 5% [v/v] ethanol). Assay samples and corresponding controls (*i.e.*, containing no WssI^ΔN^ and/or substrate) were run in quadruplicate, and the progress of the reaction was monitored at 405 nm (Cytation5) for 30 min in 1 min intervals following a 5 s initial linear shake to mix contents thoroughly. These conditions were deemed standard assay conditions for subsequent trials except where modifications are noted. The activity assay to determine optimal pH and buffer type was performed with 2-(*N*-morpholino)ethanesulfonic acid over pH ranges of 5.5 to 6.5, sodium phosphate for pH 6.5 to 7.5, and Tris for the final range of 7.5 to 9.25. In addition, standard assay conditions were modified by incorporating various inhibitors and common reducing agents to determine effects on WssI^ΔN^ activity. *Pf*WssI^ΔN^ at a concentration of 3 μM and *Ai*WssI^ΔN^ at a concentration of 2 μM were incubated for 1 h in PMSF (1 mM), MSF (5 mM), EDTA (5 mM), E-64 (0.1 mM), or pepstatin A (20 μM) in replicates of at least four. To test the effects of the absence of DTT, trials were conducted where DTT was removed from all purification and dialysis buffers, unless mentioned otherwise. Data are either reported in specific activity as units of esterase activity (*i.e.*, the amount of enzyme required to release 1 μmol of *p*-nitrophenol per min^−1^ per mg of protein^−1^) or as percent residual activity when compared with the WssI^ΔN^ controls (*i.e.*, containing no inhibitors).

The fluorescence substrate, MU-Ac, was also tested as a substrate of WssI^ΔN^ for its amenability to the planned high-throughput analyses (see later). Reaction setups were exactly the same as that for *p*NP-Ac, with the exceptions of the assay buffer (50 mM sodium phosphate, 150 mM NaCl, 1 mM DTT, pH 7), MU-Ac as the acetyl donor (final concentration of 0.5 mM along with 5% [v/v] dimethyl sulfoxide, as this was the solvent for MU-Ac), and the assay was performed in fluorescence compatible microplates (Grenier BioOne) and monitored with excitation and emission wavelengths of 365 and 450 nm, respectively for 60 min.

### Kinetic analyses

Kinetic assays were performed using the same standard assay conditions for esterase reactions outlined previously, but with acetyl donor *p*NP-Ac concentrations ranging from 0 to 10 mM and MU-Ac concentrations between 0 and 8 mM. Michaelis–Menten parameters, such as *K*_*M*_ and *k*_cat_, were calculated from the kinetic curves according to a nonlinear regression analysis of plots of initial velocity as a function of *p*NP-Ac or MU-Ac concentration using GraphPad Prism, version 8.4.3 (GraphPad Sofware, Inc).

### Avicel binding assay

To assess the ability of WssI^**ΔN**^ to bind cellulose, an Avicel binding assay (or pull-down assay) was performed in combination with SDS-PAGE for detection. Reactions contained either *Ai*WssI^ΔN^ or *Pf*WssI^ΔN^ (40 μM) and a range of quantities of Avicel (0–4 mg/ml). The enzyme binding assays and corresponding control assays, including a WssI^ΔN^ control (*i.e.*, containing no substrate), a substrate control (*i.e.*, containing no protein), and a negative protein control (*i.e.*, conducted with 40 μM DNase I), were performed in 50 mM sodium phosphate buffer (pH 7) with 150 mM sodium chloride at 22 °C for 18 h with shaking at 200 rpm on an orbital shaker. Following incubation, assays were subjected to centrifugation (21,000*g* for 5 min); thereby, separating the Avicel pellet from the supernatant. The Avicel pellet was vortexed briefly, washed with 50 mM sodium phosphate buffer, and subjected to centrifugation under the same conditions at least twice to ensure stringency of binding. The final pellet, along with supernatant and wash samples collected throughout the procedure, was suspended in SDS sample buffer and heated for 5 min at approximately 85 °C prior to electrophoresis. The supernatant and various wash samples were analyzed by SDS-PAGE on 12% (w/v) acrylamide gels according to Laemmli (89) methods with Coomassie brilliant blue staining. Relative intensity values were analyzed using ImageJ (https://imagej.nih.gov/ij/) with the plot lanes tool, and the intensity of the 37 kDa band was used to standardize intensities between different gels since the amount of molecular weight ladder used was kept constant. All statistical analyses were performed using GraphPad Prism, version 8.4.3.

### Functional analysis of acetyltransferase activity

Acetyltransfer by WssI was performed under the same esterase assay protocol outlined previously, but with the addition of cellulose or chitin oligosaccharides (1 mM) as the acceptor in the presence of either *p*NP-Ac (6 mM) or acetyl-CoA (4 mM) as the acetyl donor (with a 20–24 h reaction time). Assessment of acetyltransfer was first monitored indirectly (absorbance at 405 nm wavelength) by observing differences in *p*NP liberation rates in the presence and absence of the polysaccharide acceptors. Later to confirm the acetylated products, reactions performed with *p*NP-Ac and acceptor polysaccharides were subjected to solid-phase extraction of the carbohydrate reaction products followed by detection with MS (as outlined later). Reactions utilizing acetyl-CoA as the donor were subjected to LC–MS analyses. All model fitting and statistical analyses were performed using GraphPad Prism, version 8.4.3.

### Solid-phase extraction

Purification of reaction products using solid-phase extraction cartridges (Carbograph SPE) was conducted by the method described previously by Sychantha *et al.* (60). Briefly, the Carbograph columns were charged with 1 CV of 100% (v/v) acetonitrile and equilibrated with 2 CVs of water prior to sample application. After sample adsorption into the graphite carbon filter, the columns were washed with 2 CVs of water and 1 CV of 50% (v/v) acetonitrile followed by 100% (v/v) acetonitrile to elute the product.

### MS

The purified acetylated cellulose samples and negative controls eluted in 50% (v/v) acetonitrile (purified *via* Carbograph columns) were sent to the Advanced Analysis Centre (University of Guelph). The samples from *p*NP-Ac reactions were subjected to electrospray ionization with positive ion polarity using the Bruker AmaZon SL mass spectrometer, whereas LC–MS analyses of samples from acetyl-CoA reactions were performed on an Agilent 1200 HPLC liquid chromatograph interfaced with an Agilent UHD 6530 Q-Tof mass spectrometer. A C18 column (Agilent Extend-C18 50 mm × 2.1 mm 1.8 μm) was used for chromatographic separation with the following solvents; water with 0.1% (v/v) formic acid (A) and acetonitrile with 0.1% (v/v) formic acid (B). The mobile phase gradient consisted of initial conditions where 10% B was held for 1 min and then increased to 100% B in 29 min, followed by a column wash at 100% B for 5 min and 20 min re-equilibration. The flow rate was maintained at 0.4 ml/min throughout the gradient. The mass spectrometer electrospray capillary voltage was maintained at 4.0 Kv, and the drying gas temperature was maintained at 250 °C with a flow rate of 8 l/min. Nebulizer pressure was 30 psi, and the fragmentor was set to 160. Nozzle, skimmer, and octupole RF voltages were set at 1000, 65, and 750 V, respectively. Nitrogen (purity >99%) was used as both nebulizing, drying, and collision gas. The mass-to-charge ratio was scanned across the *m/z* range of 50 to 1500 *m/z* in 4 GHz (extended dynamic range) positive and negative ion modes. Data were collected by data-independent MS/MS acquisition with an MS and MS/MS scan rate of 1.41 spectra/s. The acquisition rate was set at 2 spectra/s. The mass axis was calibrated using the Agilent tuning mix HP0321 (Agilent Technologies) prepared in acetonitrile. Mass spectrometer control, data acquisition, and data analysis were performed with MassHunter Workstation software (B.04.00). Each spectra were analyzed for the presence of a cellopentaose peak (828 Da) or cellohexaose peak (990 Da) with the positive ion adducts of sodium (23 Da), potassium (39 Da), and/or an acetyl adduct (42 Da derivatives).

### NMR spectroscopy

For the detection of reaction products by NMR, reactions were set up in a 50 mM sodium phosphate-buffered (pH 7) solution, with 20 μM *Ai*WssI^ΔN^ or *Pf*WssI^ΔN^, 3 mM cellopentaose, and were initiated by the addition of MU-Ac (dissolved in 5% [v/v] ethanol) to a final concentration of 2 mM and allowed to proceed for 2 h at 22 °C. Corresponding controls (*i.e.*, containing no enzyme) were also prepared for comparison to the enzyme-treated samples to ensure there was no background acetylation of the oligomers. The addition of cellulase was also used in a tandem assay to digest the cellopentaose products into monomer units in an attempt to improve acetyl-product detection by NMR. For these assays, initial acetylation reactions were prepared as noted previously and allowed to proceed for 4 h prior to adding 17.5 units of a cellulase cocktail from *Trichoderma reesi* (Sigma C2730; cellulase complexes containing a β-glucosidase) and allowing the reaction to proceed for another 2 h at 22 °C. Following incubation, all reaction samples were subjected to lyophilization using a Labconco Freezone Freeze Dry System (Model 7522900) with temperature and pressure ranges between −35 and −40 °C and 133 × 10^−3^ and 350 × 10^−3^ mBar, respectively.

NMR experiments were performed at the University of Guelph NMR Centre. The lyophilized samples were dissolved in 600 μl D_2_O and transferred to a 5 mm NMR tube. ^1^H NMR spectra were recorded at 298 ± 1 K on a 600 MHz Bruker AVANCE III spectrometer equipped with a 5 mm TCI cryoprobe, with 128 scans per experiment, an acquisition time of 3.0 s, and a relaxation delay of 5.0 s. Spectra were referenced by substitution to DSS-*d6* in D_2_O (δ ^1^H_DSS_ = 0.0 ppm). The degree of monoacetylation was calculated using the following equation.

$$D_{Ac}=\frac{A_{acetyl}}{A_{anomeric}}÷\frac{3}{5}$$

In this equation, $A_{acetyl}$ and $A_{anomeric}$ are the sums of the acetyl and anomeric proton peak areas, respectively, the factor of 3 accounts for the stoichiometry of protons in an acetyl group, and the factor of 5 accounts for the stoichiometry of protons in cellopentaose. Accordingly, the aforementioned equation assumes that each cellopentaose molecule is not acetylated more than once.

### CD spectroscopy

Spectra were collected using an AVIV 215 spectrapolarimeter. Far UV spectra were recorded for protein concentrations of 3 μM for *Ai*WssI^ΔN^ or *Pf*WssI^ΔN^ in 20 mM sodium phosphate-buffered (pH 7) solution containing 30 mM NaCl. Secondary structure comparison in the presence of DTT was conducted by preparing the same samples, but with the addition of 1 mM DTT. The path length was 0.1 cm, and an internal temperature of 25 °C was maintained. The spectra are an average of four data accumulations, with 0.5 nm resolution and a range of 260 to 195 nm. Further analysis of the spectra was performed with DICHROWEB software (http://www.cryst.bbk.ac.uk/cdweb/html/home.html).

### HTS for inhibitors of WssI esterase activity

The MU-Ac fluorescence assay (described previously) was used to determine the fitness of *Ai*WssI^ΔN^ esterase activity during HTS testing. Data extrapolated as a *Zʹ* factor were the parameter of assessment for suitable WssI^ΔN^ activity in a high-throughput format. *Zʹ* values were calculated using the following equation (90):

$$\left(1-3\left(\frac{\mathrm{Sample}\;\mathrm{Standard}\;\mathrm{Deviation}-\mathrm{Blank}\;\mathrm{Standard}\;\mathrm{Deviation}}{\mathrm{Sample}\;\mathrm{Average}+\mathrm{Blank}\;\mathrm{Average}}\right)\right)$$

Large-scale HTS was conducted at the SPARC BioCentre Drug Discovery (Hospital for Sick Children, Toronto, Canada) with a subset of the Chembridge DIVERset library collection (as well as 135 manually selected compounds based on success against other SGNH enzymes) in a 384-well format. The assay was performed in singlet during the primary screen of the entire compound list and in triplicate for a follow-up confirmatory screen. Reaction setups for both screens were conducted with 50 mM sodium phosphate buffer (pH 6.5), 3 μM *Ai*WssI^ΔN^ and 0.5 mM MU-Ac (final concentration of 5% [v/v] dimethyl sulfoxide) using a Thermo MultiDrop Combi Reagent Dispenser. The reaction was initiated by the addition of 20 μM of the test compounds using an Echo 550 acoustic dispenser (Labcyte). Fluorescence was monitored with a SynergyNeo microplate reader (BioTek) using excitation and emission wavelengths of 365 and 450 nm, respectively, for approximately 45 min alongside appropriate negative and positive controls. Data analysis was conducted using CDD Vault Analysis Software (Collaborative Drug Discovery, Inc), and hits were identified as compounds that had a Z-score ≤−3. The hits remaining after the primary and confirmatory screening were examined further to identify possible PAINS, which could result in false-positive results (91). Further refinement of the lead candidates was also conducted by assessing the fluorescence quenching potential of lead compound hits by assaying the quenching potential of different compound concentrations (10.1 μM and/or 50.5 μM) against the fluorescence of 4-methylumbeliferone (247 μM) to reveal any potential false positives that were not actually because of *Ai*WssI^ΔN^ activity. All fluorescence readings were normalized using the following equation to determine the percentage of quenching (92):

$$Normalized\;Flouresence=\frac{F_{obs}-\mu_{Fmin}}{\mu_{Fmax}-\mu_{Fmin}}\;x\;100\%$$

In the aforementioned formula, *F*_*obs*_ is the sample fluorescence value, *μ*_*Fmin*_ is the mean fluorescence value of the negative controls, and *μ*_*Fmax*_ is the mean fluorescence value of the positive controls.

Dose–response assays of the remaining selected target compounds were then conducted across concentrations ranging from 0 to 215 μM to determine IC_50_ values for each candidate against *Ai*WssI^ΔN^ with MU-Ac in HTS format and standard assay format (noted previously). All inhibitor compounds for confirmatory assays were purchased from Enamine Ltd, with the exception of compound 1 (Santa Cruz Biotechnology, Inc), Compounds 2 and 7 (Molport *via* Maybridge Ltd and Vitas M Chemical Ltd, respectively) and compound 6 (Mcule, Inc). The dose–response curves were generated using either CDD Vault Analysis Software (Collaborative Drug Discovery, Inc) or GraphPad Prism, version 8.4.3. Inhibition constants (*K*_i_) were calculated using the Cheng–Prusoff equation for competitive inhibition that is calculated using the *K*_*M*_ of the uninhibited enzyme, the substrate concentration (S) at which the IC_50_ values were calculated, and the IC_50_ value of the inhibitor (92):

$$K_{i}=\frac{{IC}_{50}}{\left({S/K}_{M}+1\right)}$$

## Data availability

The GenBank accession numbers for *Ai*WssI and *Pf*WssI used for sequences in this study are AFRQ01000037.1 and WP_012721729, respectively. The raw NMR and MS data have been deposited in FigShare (10.6084/m9.figshare.23509230 and 10.6084/m9.figshare.23509221, respectively). All other data are contained within the article or supporting information.

## Supporting information

This article contains supporting information.

## Conflict of interest

The authors declare that they have no conflicts of interest with the contents of this article.

## References

[1] Karygianni L., Ren Z., Koo H., Thurnheer T. (2020). Biofilm matrixome: extracellular components in structured microbial communities. Trends Microbiol..

[2] Costerton J.W., Stewart P.S., Greenberg E.P. (1999). Bacterial biofilms: a common cause of persistent infections. Science.

[3] Yaron S., Römling U. (2014). Biofilm formation by enteric pathogens and its role in plant colonization and persistence. Microb. Biotechnol..

[4] Lynch A.S., Robertson G.T. (2008). Bacterial and fungal biofilm infections. Annu. Rev. Med..

[5] Aly M.A., Reimhult E., Kneifel W., Domig K.J. (2018). Characterization of biofilm formation by *Cronobacter* spp. isolates of different food origin under model conditions. J. Food Prot..

[6] Canale-Parola E., Borasky R., Wolfe R.S. (1961). Studies on *Sarcina ventriculi* III. Localization of cellulose. J. Bacteriol..

[7] Fux C.A., Costerton J.W., Stewart P.S., Stoodley P. (2005). Survival strategies of infectious biofilms. Trends Microbiol..

[8] Flemming H.-C., Wingender J., Szewzyk U., Steinberg P., Rice S.A., Kjelleberg S. (2016). Biofilms: an emergent form of bacterial life. Nat. Rev. Microbiol..

[9] Epstein A.K., Pokroy B., Seminara A., Aizenberg J. (2011). Bacterial biofilm shows persistent resistance to liquid wetting and gas penetration. Proc. Natl. Acad. Sci. U. S. A..

[10] Whitfield G.B., Marmont L.S., Howell P.L. (2015). Enzymatic modifications of exopolysaccharides enhance bacterial persistence. Front. Microbiol..

[11] Ross P., Mayer R., Benziman M. (1991). Cellulose biosynthesis and function in bacteria. Microbiol. Rev..

[12] Szymczak I., Pietrzyk-Brzezińska A.J., Duszyński K., Ryngajłło M. (2022). Characterization of the putative acylated cellulose synthase operon in *Komagataeibacter xylinus* E25. Int. J. Mol. Sci..

[13] McLaughlin K., Folorunso A.O., Deeni Y.Y., Foster D., Gorbatiuk O., Hapca S.M. (2017). Biofilm formation and cellulose expression by *Bordetella avium* 197N, the causative agent of bordetellosis in birds and an opportunistic respiratory pathogen in humans. Res. Microbiol..

[14] Saldaña Z., Xicohtencatl-Cortes J., Avelino F., Phillips A.D., Kaper J.B., Puente J.L. (2009). Synergistic role of curli and cellulose in cell adherence and biofilm formation of attaching and effacing *Escherichia coli* and identification of Fis as a negative regulator of curli. Environ. Microbiol..

[15] Anderson A.C., Burnett A.J.N., Hiscock L., Maly K.E., Weadge J.T. (2020). The *Escherichia coli* cellulose synthase subunit G (BcsG) is a Zn2+-dependent phosphoethanolamine transferase. J. Biol. Chem..

[16] Omadjela O., Narahari A., Strumillo J., Mélida H., Mazur O., Bulone V. (2013). BcsA and BcsB form the catalytically active core of bacterial cellulose synthase sufficient for *in vitro* cellulose synthesis. Proc. Natl. Acad. Sci. U. S. A..

[17] Acheson J.F., Ho R., Goularte N.F., Cegelski L., Zimmer J. (2021). Molecular organization of the *E. coli* cellulose synthase macrocomplex. Nat. Struct. Mol. Bio..

[18] Spiers A.J., Bohannon J., Gehrig S.M., Rainey P.B. (2003). Biofilm formation at the air-liquid interface by the *Pseudomonas fluorescens* SBW25 wrinkly spreader requires an acetylated form of cellulose. Mol. Microbiol..

[19] Römling U., Galperin M.Y. (2015). Bacterial cellulose biosynthesis: diversity of operons, subunits, products, and functions. Trends Microbiol..

[20] Thongsomboon W., Serra D.O., Possling A., Hadjineophytou C., Hengge R., Cegelski L. (2018). Phosphoethanolamine cellulose: a naturally produced chemically modified cellulose. Science.

[21] Thongsomboon W., Werby S.H., Cegelski L. (2020). Evaluation of phosphoethanolamine cellulose production among bacterial communities using Congo red fluorescence. J. Bacteriol..

[22] Anderson A.C., Burnett A.J.N., Constable S., Hiscock L., Maly K.E., Weadge J.T. (2021). A mechanistic basis for phosphoethanolamine modification of the cellulose biofilm matrix in *Escherichia coli*. Biochemistry.

[23] Bundalovic-Torma C., Whitfield G.B., Marmont L.S., Howell P.L., Parkinson J. (2020). A systematic pipeline for classifying bacterial operons reveals the evolutionary landscape of biofilm machineries. PLoS Comput. Biol..

[24] Kobayashi H. (2005). Airway biofilms: implications for pathogenesis and therapy of respiratory tract infections. Treat. Respir. Med..

[25] Franklin M.J., Nivens D.E., Weadge J.T., Lynne Howell P. (2011). Biosynthesis of the *Pseudomonas aeruginosa* extracellular polysaccharides, alginate, Pel, and Psl. Front. Microbiol..

[26] Dickson R.P., Erb-Downward J.R., Freeman C.M., Walker N., Scales B.S., Beck J.M. (2014). Changes in the lung microbiome following lung transplantation include the emergence of two distinct *Pseudomonas* species with distinct clinical associations. PLoS One.

[27] Chen S., Zhu Q., Xiao Y., Wu C., Jiang Z., Liu L. (2021). Clinical and etiological analysis of co-infections and secondary infections in COVID-19 patients: an observational study. Clin. Respir. J..

[28] Scales B.S., Dickson R.P., LiPuma J.J., Huffnagle G.B. (2014). Microbiology, genomics, and clinical significance of the *Pseudomonas fluorescens* species complex, an unappreciated colonizer of humans. Clin. Microbiol. Rev..

[29] Meliani A., Bensoltane A. (2015). Review of *Pseudomonas* attachment and biofilm formation in food industry. Poult. Fish. Wildl. Sci..

[30] Deinema M.H., Zevenhuizen L.P.T.M. (1971). Formation of cellulose fibrils by gram-negative bacteria and their role in bacterial flocculation. Arch. Mikrobiol..

[31] Kidd T.J., Ramsay K.A., Hu H., Bye P.T.P., Elkins M.R., Grimwood K. (2009). Low rates of *Pseudomonas aeruginosa* misidentification in isolates from cystic fibrosis patients. J. Clin. Microbiol..

[32] Pérez Barragán E., Sandino Pérez J., Corbella L., Orellana M.A., Fernández-Ruiz M. (2018). *Achromobacter xylosoxidans* bacteremia: clinical and microbiological features in a 10-year case series. Rev. Esp. Quimioter..

[33] Konstantinović N., Ćirković I., Đukić S., Marić V., Božić D. (2017). Biofilm formation of *Achromobacter xylosoxidans* on contact lens. Acta Microbiol. Immunol. Hung..

[34] Dupont C., Michon A.-L., Jumas-Bilak E., Nørskov-Lauritsen N., Chiron R., Marchandin H. (2015). Intrapatient diversity of *Achromobacter* spp. involved in chronic colonization of Cystic Fibrosis airways. Infect. Genet. Evol..

[35] Nielsen S.M., Nørskov-Lauritsen N., Bjarnsholt T., Meyer R.L. (2016). *Achromobacter* species isolated from cystic fibrosis patients reveal distinctly different biofilm morphotypes. Microorganisms.

[36] Filipic B., Malesevic M., Vasiljevic Z., Lukic J., Novovic K., Kojic M. (2017). Uncovering differences in virulence markers associated with *Achromobacter* species of CF and non-CF origin. Front. Cell. Infect. Microbiol..

[37] Trancassini M., Iebba V., Citerà N., Tuccio V., Magni A., Varesi P. (2014). Outbreak of *Achromobacter xylosoxidans* in an Italian cystic fibrosis center: genome variability, biofilm production, antibiotic resistance, and motility in isolated strains. Front. Microbiol..

[38] Amoureux L., Bador J., Verrier T., Mjahed H., De Curraize C., Neuwirth C. (2016). *Achromobacter xylosoxidans* is the predominant *Achromobacter* species isolated from diverse non-respiratory samples. Epidemiol. Infect..

[39] Park J.H., Song N.H., Koh J.W. (2012). *Achromobacter xylosoxidans* keratitis after contact lens usage. Korean J. Ophthalmol..

[40] Amoureux L., Bador J., Fardeheb S., Mabille C., Couchot C., Massip C. (2013). Detection of *Achromobacter xylosoxidans* in hospital, domestic, and outdoor environmental samples and comparison with human clinical isolates. Appl. Environ. Microbiol..

[41] Moretti M., Gilardi G., Gullino M.U., Garibaldi A. (2008). Biological control potential of *Achromobacter xylosoxydans* for suppressing *Fusarium* wilt of tomato. Int. J. Bot..

[42] Haas D., Keel C. (2003). Regulation of antibiotic production in root-colonizing *Peudomonas* spp. and relevance for biological control of plant disease. Annu. Rev. Phytopathol..

[43] Rainey P.B., Bailey M.J. (1996). Physical and genetic map of the *Pseudomonas fluorescens* SBW25 chromosome. Mol. Microbiol..

[44] Rainey P.B., Travisano M. (1998). Adaptive radiation in a heterogeneous environment. Nature.

[45] Rainey P.B. (1999). Adaptation of *Pseudomonas fluorescens* to the plant rhizosphere. Environ. Microbiol..

[46] Spiers A.J., Kahn S.G., Bohannon J., Travisano M., Rainey P.B. (2002). Adaptive divergence in experimental populations of *Pseudomonas fluorescens*. I. Genetic and phenotypic bases of wrinkly spreader fitness. Genetics.

[47] Gal M., Preston G.M., Massey R.C., Spiers A.J., Rainey P.B. (2003). Genes encoding a cellulosic polymer contribute toward the ecological success of *Pseudomonas fluorescens* SBW25 on plant surfaces. Mol. Ecol..

[48] Spiers A.J. (2014). A mechanistic explanation linking adaptive mutation, niche change, and fitness advantage for the wrinkly spreader. Int. J. Evol. Biol..

[49] Koza A., Kusmierska A., McLaughlin K., Moshynets O., Spiers A.J. (2017). Adaptive radiation of *Pseudomonas fluorescens* SBW25 in experimental microcosms provides an understanding of the evolutionary ecology and molecular biology of A-L interface biofilm formation. FEMS Microbiol. Lett..

[50] Whitney J.C., Howell P.L. (2013). Synthase-dependent exopolysaccharide secretion in Gram-negative bacteria. Trends Microbiol..

[51] Morgan J.L.W., McNamara J.T., Zimmer J. (2014). Mechanism of activation of bacterial cellulose synthase by cyclic di-GMP. Nat. Struct. Mol. Biol..

[52] Low K.E., Howell P.L. (2018). Gram-negative synthase-dependent exopolysaccharide biosynthetic machines. Curr. Opin. Struct. Biol..

[53] Mazur O., Zimmer J. (2011). Apo- and cellopentaose-bound structures of the bacterial cellulose synthase subunit BcsZ. J. Biol. Chem..

[54] Scott W., Lowrance B., Anderson A.C., Weadge J.T. (2020). Identification of the clostridial cellulose synthase and characterization of the cognate glycosyl hydrolase, CcsZ. PLoS One.

[55] Umeda Y., Hirano A., Ishibashi M., Akiyama H., Onizuka T., Ikeuchi M. (1999). Cloning of cellulose synthase genes from *Acetobacter xylinum* JCM 7664: Implication of a novel set of cellulose synthase genes. DNA Res..

[56] Le Quéré B., Ghigo J.-M. (2009). BcsQ is an essential component of the *Escherichia coli* cellulose biosynthesis apparatus that localizes at the bacterial cell pole. Mol. Microbiol..

[57] Ekici Ö.D., Paetzel M., Dalbey R.E. (2008). Unconventional serine proteases: variations on the catalytic Ser/His/Asp triad configuration. Protein Sci..

[58] Lee L.C., Lee Y.L., Leu R.J., Shaw J.F. (2006). Functional role of catalytic triad and oxyanion hole-forming residues on enzyme activity of *Escherichia coli* thioesterase I/protease I/phospholipase L1. Biochem. J..

[59] Moynihan P.J., Clarke A.J. (2014). Mechanism of action of peptidoglycan O-acetyltransferase B involves a Ser-His-Asp catalytic triad. Biochemistry.

[60] Sychantha D., Little D.J., Chapman R.N., Boons G.-J., Robinson H., Howell P.L. (2017). PatB1 is an O-acetyltransferase that decorates secondary cell wall polysaccharides. Nat. Chem. Biol..

[61] Baker P., Ricer T., Moynihan P.J., Kitova E.N., Walvoort M.T.C., Little D.J. (2014). *P. aeruginosa* SGNH hydrolase-like proteins AlgJ and AlgX have similar topology but separate and distinct roles in alginate acetylation. PLoS Pathog..

[62] Riley L.M., Weadge J.T., Baker P., Robinson H., Codée J.D.C., Tipton P.A. (2013). Structural and functional characterization of *Pseudomonas aeruginosa* AlgX: role of Algx in alginate acetylation. J. Biol. Chem..

[63] Akoh C.C., Lee G.-C., Liaw Y.-C., Huang T.-H., Shaw J.-F. (2004). GDSL family of serine esterases/lipases. Prog. Lipid Res..

[64] Bomble Y., Lunin V., Bharadwaj V., Alahuhta P., Himmel M., Wang H.-T. (2020). Molecular mechanism of polysaccharide acetylation by the *Arabidopsis* xylan O-acetyltransferase XOAT1. Plant Cell.

[65] Weadge J.T., Clarke A.J. (2006). Identification and characterization of O-acetylpeptidoglycan esterase: a novel enzyme discovered in *Neisseria gonorrhoeae*. Biochemistry.

[66] Sychantha D., Clarke A.J. (2018). Peptidoglycan modification by the catalytic domain of *Streptococcus pneumoniae* OatA follows a ping-pong bi-bi mechanism of action. Biochemistry.

[67] Moynihan P.J., Clarke A.J. (2014). Substrate specificity and kinetic characterization of peptidoglycan O-acetyltransferase B from *Neisseria gonorrhoeae*. J. Biol. Chem..

[68] Wilks J.C., Slonczewski J.L. (2007). pH of the cytoplasm and periplasm of *Escherichia coli*: rapid measurement by green fluorescent protein fluorimetry. J. Bacteriol..

[69] Weadge J.T., Clarke A.J. (2007). *Neisseria gonorrheae* O-acetylpeptidoglycan esterase, a serine esterase with a Ser-His-Asp catalytic triad. Biochemistry.

[70] Sychantha D., Jones C.S., Little D.J., Moynihan P.J., Robinson H., Galley N.F. (2017). *In vitro* characterization of the antivirulence target of Gram-positive pathogens, peptidoglycan O-acetyltransferase A (OatA). PLoS Pathog..

[71] Cronan J.E., Thomas J. (2009). Bacterial fatty acid synthesis and its relationships with polyketide synthetic pathways. Methods Enzymol..

[72] Ma D., Wang Z., Merrikh C.N., Lang K.S., Lu P., Li X. (2018). Crystal structure of a membrane-bound O-acyltransferase. Nature.

[73] Wang L., Qian H., Nian Y., Han Y., Ren Z., Zhang H. (2020). Structure and mechanism of human diacylglycerol O-acyltransferase 1. Nature.

[74] Jones C.S., Anderson A.C., Clarke A.J. (2021). Mechanism of *Staphylococcus aureus* peptidoglycan O-acetyltransferase A as an O-acyltransferase. Proc. Natl. Acad. Sci. U. S. A..

[75] Myers D.K., Kemp A. (1954). Inhibition of esterases by the fluorides of organic acids. Nature.

[76] Baell J.B., Holloway G.A. (2010). New substructure filters for removal of pan assay interference compounds (PAINS) from screening libraries and for their exclusion in bioassays. J. Med. Chem..

[77] Gazit A., Yaish P., Gilon C., Levitzki A. (1989). Tyrphostins I: synthesis and biological activity of protein tyrosine kinase inhibitors. J. Med. Chem..

[78] Martin B.L. (1998). Inhibition of calcineurin by the tyrphostin class of tyrosine kinase inhibitors. Biochem. Pharmacol..

[79] Moynihan P.J., Clarke A.J. (2013). Assay for peptidoglycan O-acetyltransferase: a potential new antibacterial target. Anal. Biochem..

[80] Skjåk-Braek G., Grasdalen H., Larsen B. (1986). Monomer sequence and acetylation pattern in some bacterial alginates. Carbohydr. Res..

[81] Chanasit W., Gonzaga Z.J.C., Rehm B.H.A. (2020). Analysis of the alginate O-acetylation machinery in *Pseudomonas aeruginosa*. Appl. Microbiol. Biotechnol..

[82] Nakai K., Horton P., Nakai K., Horton P. (1999). Psort: a program for detecting sorting signals in proteins and predicting their subcellular localization. Trends Biochem. Sci..

[83] Kelley L.A., Mezulis S., Yates C.M., Wass M.N., Sternberg M.J.E. (2015). The Phyre2 web portal for protein modeling, prediction and analysis. Nat. Protoc..

[84] Jumper J., Evans R., Pritzel A., Green T., Figurnov M., Ronneberger O. (2021). Highly accurate protein structure prediction with AlphaFold. Nature.

[85] Mirdita M., Schütze K., Moriwaki Y., Heo L., Ovchinnikov S., Steinegger M. (2022). ColabFold: making protein folding accessible to all. Nat. Methods.

[86] Sievers F., Wilm A., Dineen D., Gibson T.J., Karplus K., Li W. (2011). Fast, scalable generation of high-quality protein multiple sequence alignments using clustal omega. Mol. Syst. Biol..

[87] Krogh A., Larsson B., von Heijne G., Sonnhammer E.L. (2001). Predicting transmembrane protein topology with a hidden markov model: application to complete genomes. J. Mol. Biol..

[88] Bond C.S. (2003). TopDraw: a sketchpad for protein structure topology cartoons. Bioinformatics.

[89] Laemmli U.K. (1970). Cleavage of structural proteins during the assembly of the head of bacteriophage T4. Nature.

[90] Zhang J.-H., Chung T.D.Y., Oldenburg K.R. (1999). A simple statistical parameter for use in evaluation and validation of high throughput screening assays. J. Biomol. Screen..

[91] Brott A.S., Jones C.S., Clarke A.J. (2019). Development of a high throughput screen for the identification of inhibitors of peptidoglycan O-acetyltransferases, new potential antibacterial targets. Antibiotics (Basel).

[92] Cer R.Z., Mudunuri U., Stephens R., Lebeda F.J. (2009). IC50-to-Ki: a web-based tool for converting IC50 to Ki values for inhibitors of enzyme activity and ligand binding. Nucleic Acids Res..

