# Supplementary material for: WssI from the Gram-negative bacterial cellulose synthase is an O-acetyltransferase that acts on cello-oligomers with several acetyl donor substrates
Source: J Biol Chem. 2023 May 22;299(7):104849. doi: 10.1016/j.jbc.2023.104849 (PMC10302187; doi:10.1016/j.jbc.2023.104849)
Supplement: Supporting Data [file mmc1.docx]

**Supporting Information for:**

**WssI from the Gram-Negative Bacterial Cellulose Synthase is an *O*-acetyltransferase that Acts on Cello-oligomers with Several Acetyl Donor Substrates**

## **Alysha J.N. Burnett^1^, Emily Rodriguez^1^, Shirley Constable^1^, Brian Lowrance^1^, Michael Fish^1^ and Joel T. Weadge^1^**

^1^Department of Biology, Wilfrid Laurier University, Waterloo, ON, Canada

**Corresponding author information:** Joel T. Weadge: Department of Biology, Wilfrid Laurier University, 75 University Ave W. Waterloo ON N2L 3C5; Phone: 519-884-0710 x 2161; E-mail: [jweadge@wlu.ca](mailto:jweadge@wlu.ca)

**This file includes:**

Table S1

Figures S1 to S5

Supplementary methods for NMR reactions

**Table S1:** Bacterial Strains and Plasmids Used in this Study

| **Strain/Plasmid** | **Description** | **Source** |
| --- | --- | --- |
| **Strains** |  |  |
| *E. coli* Top 10 | F- *mcrA* Δ( *mrr-hsd*RMS-*mcr*BC) Φ80*lac*ZΔM15 Δ *lac*X74 *rec*A1*ara*D139 Δ( *araleu*)7697 *gal*U *gal*K *rps*L (StrR) *end*A1 *nup*G | Invitrogen |
| *E. coli* Rosetta codon plus | *E. coli* B F– ompT hsdS(r_B_ – m_B_ –) dcm+ Tetr gal λ (DE3) endA Hte [argU ileY leuW Camr] | Agilent |
| *E. coli* BL21 (DE3) | F– *ompT hsdS*B(rB– mB–) *gal dcm* (DE3)\ | Novagen |
| **Plasmids** |  |  |
| p*Ai*WssI^ΔN^-His_6_ | pET24 plasmid vector encoding amino acids 95 to 374 of *Ai*WssI with a C-terminal His_6_ tag fusion; Kan^R^ | Genescript |
| p*Pf*WssI^ΔN^-His_6_ | pET28 plasmid vector encoding amino acids 92-374 of *Pf*WssI with a C-terminal His_6_ tag fusion; Kan^R^ | This study |

AlgX/41-347 GNLCPAAAYDSRYNTKYLGFFTHLVQAQDDWLFRTTYDLRTD---FGTSAEGWRELRALR 57

AlgJ/78-373 -----------------NEGRPGVVLGRDQWLFSDEE-FKPTAGAEQLMQENLALIRGVR 42

*Ai*WssI/96-380 -----------------ADLGPRVRRGCDGWLFLGDE-LQPHPAARENQAERARIVVSLR 42

*Pf*WssI/92-374 -----------------GDTGPRVRPGCPGWLFISDE-LRINRHAEANAQTKAQAVIDLQ 42

: . *** :: : ::

AlgX/41-347 DELKRKGIELVVVYQPTRGLVNREKLSPAEK-AGFDYELAKKNYLATIARFRQAGIWTPD 116

AlgJ/78-373 DTLQQHGSQLVLAIVPAKARVYTEYL-GKERPASLHD----DLYNQFHAQARQANVFAPD 97

*Ai*WssI/96-380 DALAARGIQLLVAVVPDKSRIESERLCGLHRSAGFED----R-LSSWVGVLRAQGVATVD 97

*Pf*WssI/92-374 KQLGQKGIDLQVVVVPDKSRIAAAQRCGLYRPAVLDN----R-VRDWTAMLQAAGVSALD 97

. * :* :* :. * :. : : * :. . : .: : *

AlgX/41-347 FSPLFDE-KEEHAYYFKGDHHWTPHGARRSAKIVAETLKQVPGFEEIPKKQFESK----- 170

AlgJ/78-373 LMAPMEQAKARGQVFLRTDTHWTPMGAEVAAQALAEAVSRQSL-LNGDPQAFITEAGNTA 156

*Ai*WssI/96-380 LSAALRG--VPQDAYYRNDSHWTEAGAGAAARAVADQVRASGV-ALQAPQRWRVTAQPPA 154

*Pf*WssI/92-374 LTETLKP--LGAEAYLRTDTHWSEIGSNAGAKAVAQRTQQRGI-KATPEQTFDITQAPLA 154

: : : : * **: *: .*: :*: : :

AlgX/41-347 -RVGLLSKLGTFHKAAAQLCGNSYATQYVDRFETEP---VGASDSGDLFGDGGNPQIALV 226

AlgJ/78-373 PYKGDLTNFLPLDPLFSNLLP---APDNLQKRTTRPVD-AEGDAGDALFADKQIP-VALV 211

*Ai*WssI/96-380 PRPGDLVRLAGVDWLPLAWQP---RAEVVALHTYAPEAAASAGDADDLFGDSALPSLALV 211

*Pf*WssI/92-374 VRPGDLVRLAGLDWLPPTLQP---PGESVAASTTH-ETGGATSNADDLFGDAGLPNVALI 210

* * .: .. : : . .. **.* * :**:

AlgX/41-347 GTSNSG-PAYNFAGFLEEFSGADILNNAVSGGGFDSSLLAYMTSEEFHKNPPKILIWEFA 285

AlgJ/78-373 GTSYSANPHWNFLGALQQALRSDVANYAEDGHGPLLPMLKYLQSDAFKNAAPQVVVWEFP 271

*Ai*WssI/96-380 GTSFSRTS--EFLPQLSQDLGVAVGNFARDGGKFGGAAQAYFKSPAWKQSPPRLLIWEMD 269

*Pf*WssI/92-374 GTSFSRNS--NFVGFLQKALNAPVGNFSKDGGEFSGAAKAYFDSPAFKQTPPKLLIWEIP 268

*** * :* *.: : * : .* *: * ::: *::::**:

AlgX/41-347 THYDMAQKSF---YRQAMPLVDNGC 307

AlgJ/78-373 ERYLPMKNDLSSFDPQWIAQLKNSR 296

*Ai*WssI/96-380 ERDLGAPLAAEDRVGF--------- 285

*Pf*WssI/92-374 ERDLQTPYDVITIGQ---------- 283

:

**Figure S1:** Clustal Omega Sequence Alignment of PfWssI^ΔN^, AiWssI^ΔN^, AlgJ and AlgX. The sequence range covers the acetyltransferase domain of each of these proteins and conserved Asp (D), His (H) and Ser (S) residues are highlighted in gray.

**

**Figure S2.** Far-UV CD and Tryptophan fluorescence spectroscopy results of AiWssI^ΔN^ and PfWssI^ΔN^.

Far-UV CD spectra of (a) AiWssI^ΔN^ and (b) PfWssI^ΔN^ (3 µM) in the absence and presence of DTT (1 mM) were measured at 25 ºC from 195-260 nm. (Inset) Secondary structure content of each protein in the presence and absence of DTT was estimated from backbone CD spectra using the deconvolution program DichroWeb. Fluorescence spectra of (c) AiWssI^ΔN^ or (d) PfWssI^ΔN^ (3 µM) were collected in the absence and presence of DTT (1 mM) by measuring at 25 ºC with an excitation wavelength of 280 nm and emission wavelengths from 300-500 nm. In all cases the spectra with and without DTT overlap, indicating that reduction of cysteine residues by DTT does not lead to detectable secondary structure changes under these test conditions. The difference in fluorescence signal response between the WssI^ΔN^ proteins is most likely due to the fact that AiWssI^ΔN^ contains eight tryptophan residues compared to five in PfWssI^ΔN^.

**50**

**37**

**25**

**20**

**15**

**10**

***1 2 3 4 5 6 7 8 9***





**Figure S3.** Representative Purification of PfWssI^ΔN^.

Immobilized metal affinity chromatography (IMAC) and anion exchange chromatography purification fractions of PfWssI^ΔN^ resolved on a Coomassie Brilliant Blue stained SDS-polyacrylamide gel. The expected molecular mass of C-terminal His_6_-tag PfWssI^ΔN^ is 31.5 kDa. Lane 1, molecular mass marker (kDa); Lane 2, cell lysate; Lane 3, IMAC unbound proteins; Lane 4, IMAC buffer wash (50 mM imidazole); Lane 5, IMAC elution (175 mM imidazole); Lane 7, anion exchange unbound protein; Lane 8, anion exchange wash (125 mM NaCl); Lane 9, anion exchange elution of PfWssI^ΔN^ (200 mM NaCl). The purification of AiWssI^ΔN^ was highly similar to that shown for PfWssI^ΔN^ here.

**

**Figure S4.** Specific activity of AiWssI^ΔN^ and PfWssI^ΔN^ in the presence of chito-oligosaccharide acceptors.

Scatter plots representing specific activity trends of AiWssI^ΔN^ (a) and PfWssI^ΔN^ (b) against chitin oligosaccharides (DP 2 to 6). Reaction rates monitoring the cleavage of pNP-Ac at 405 nm in the presence of a chitin acceptor were measured. The reactions were conducted in 50 mM sodium phosphate buffer (pH 7), 1 mM oligosaccharide, 3 µM (PfWssI^ΔN^) or 2 µM (AiWssI^ΔN^) enzyme and 6 mM pNP-Ac (replicates of 4) for 30 min at 23 $℃$. None of the trials were significantly different from the no acceptor control (t-test, p > 0.05).

(a)

(b)

**Figure S****5.** ^1^H DOSY NMR spectrum of reaction products by *Ai*WssI^ΔN^ with cellopentaose in D_2_O. (a) Full ^1^H DOSY spectra. The ^1^H chemical shift (horizontal axis) is plotted against the translational diffusion coefficient in m^2^/s (log scale) (vertical axis). Peaks belonging to larger, more slowly diffusing molecules appear near the top of the plot, while peaks belonging to smaller, more quickly diffusing molecules appear near the bottom of the plot. The cellopentaose ring protons (between 5.5 ppm and 3.0 ppm) have a diffusion coefficient near -9.55 log(m^2^/s). (b) Expansion of the acetyl region of the spectra. The five acetyl groups between 2.12 and 2.20 ppm all share the same translation diffusion coefficient as the ring protons (highlighted red area in both panels a and b), consistent with acetyl groups covalently bonded to cellopentaose.

**Figure S6.** Representative HTS Assay Compatibility Analysis. The % residual activity of the AiWssI^ΔN^-containing positive reactions (black circles) and negative control reactions (open circles) are plotted. A Zʹ of 0.63 was calculated for this data subset of an AiWssI^ΔN^ purification and an average Zʹ of 0.57 was maintained across HTS analyses (>60 test plates).

**Supplementary Methods.**

**NMR Reactions.** DOSY spectra were recorded using the *ledbpgp2s* pulse sequence provided with Bruker TopSpin 3.6.5. For the BPP-LED (bipolar pulse pair – longitudinal-eddy-current delay) experiment ([J Mag Res A, 115, 260-264](https://doi.org/10.1006/jmra.1995.1176)) the NMR signal intensity (I) is a function of the molecular diffusion coefficient (D) related by the following equation.

I(g) = I(0) exp[-D(γgδ)^2^(Δ-δ/3-τ/2)]

Within the above equation, g is the strength of each gradient pulse, γ is the ^1^H gyromagnetic ratio, δ/2 is the duration of each gradient pulse, Δ is the delay between the so-called ‘encoding’ and ‘decoding’ gradients, and τ is the gradient stabilization delay. In this work, δ/2 was fixed at 1.4 msec, Δ was held fixed at 80 msec, and τ was fixed at 0.2 msec, while g was varied quadratically in 16 steps from 2.39 to 45.3 G/cm (accounting for the smoothed sine-bell amplitude profile of the gradient pulses). The LED time was set to 5 msec. Eight transients were collected at each gradient strength, with a 5 s relaxation delay between scans, for a total experiment time of roughly 14 min. The DOSY spectrum was processed in Bruker TopSpin 4.1.4 by optimizing a single diffusion component at each chemical shift value.

***Fluorescence Spectroscopy.*** Spectra were collected using a Cary Eclipse spectrofluorometer.

The fluorescence was recorded for protein concentrations of 3 µM for *Ai*WssI^ΔN^ or *Pf*WssI^ΔN^ in 20

mM sodium phosphate buffered (pH 7) solution containing 30 mM NaCl. Conformational changes

(as assessed by differences in aromatic residue buried content fluorescence) were monitored in the presence of DTT by preparing the same samples, but with the addition of 1 mM DTT. All reactions were measured at 25 ºC with an excitation wavelength of 280 nm and emission wavelengths from 300-500 nm. Measurements were carried out in 1 cm path-length quartz cuvettes at 1 nm resolution.
